# Resistance training for depression: a systematic review and meta-analysis of randomized controlled trials

**DOI:** 10.3389/fpsyg.2025.1655855

**Published:** 2025-12-15

**Authors:** Yuanbo Chang, Hai Wang, Xinbi Zhang, Shun Shan, Haiyuan Liu

**Affiliations:** 1 Capital University of Physical Education and Sports, Beijing, China; 2 Institute of Physical Education, Hunan First Normal University, Changsha, Hunan, China

**Keywords:** resistance training, depression, exercise, meta-analysis, randomized controlled trial

## Abstract

**Introduction:**

Depression is a prevalent and disabling mental disorder worldwide. Resistance training (RT) has emerged as a promising adjunct intervention, but comprehensive quantitative synthesis on its efficacy and optimal exercise prescription remains limited.

**Objective:**

To evaluate the effects of RT on depressive symptoms in adults with a clinically diagnosed depressive disorder, and to explore—exploratorily—whether participant characteristics and prescription components modify outcomes.

**Methods:**

We searched PubMed, Embase, Web of Science, Cochrane Central, and CNKI from inception through August 2024 for randomized controlled trials (RCTs) comparing RT to a non-exercise control in adults with depression (PROSPERO CRD42024583413). Two reviewers independently screened studies and extracted data in accordance with PRISMA 2020. Depression outcomes were pooled as standardized mean differences (SMDs) with 95% confidence intervals (CIs) using a random-effects model, with unit-of-analysis safeguards for multi-arm trials. Pre-specified exploratory analyses evaluated potential effect modifiers (e.g., clinical phenotype [primary vs. comorbid], training frequency, age, baseline severity, duration, intensity, weekly volume). Risk of bias was assessed by two independent reviewers using the Cochrane tool; publication bias was evaluated by funnel plot and Begg’s test, noting limited power with this study count. Sensitivity analyses included exclusion of high-risk studies and leave-one-out influence checks to test robustness.

**Results:**

Twenty-nine RCTs (*N* = 2,036) met inclusion criteria. RT significantly reduced depressive symptoms compared to controls (pooled SMD = −0.94, 95% CI: −1.16 to −0.72, *p* < 0.001), though heterogeneity was high (*I*^2^ ≈ 80%). Benefits were observed in both primary depressive disorder (SMD − 1.12, 95% CI − 1.43 to −0.81) and comorbid depression (SMD − 0.66, −0.96 to −0.36), with a modest between-subgroup contrast (Q_between = 4.41, *p* = 0.036). Effects were directionally consistent across self-report and observer-rated measures and across frequency strata (<3 vs. ≥ 3 sessions/week), with no compelling between-subgroup differences; beyond these key strata, exploratory subgroup analyses across age, baseline severity, duration, intensity, and weekly volume likewise did not reveal consistent between-group differences, and estimates were imprecise in small strata. Sensitivity analyses—excluding high-risk studies and via leave-one-out influence checks—yielded estimates of similar magnitude. The funnel plot appeared broadly symmetric and Begg’s test was non-significant, while acknowledging limited power with this study count.

**Conclusion:**

RT meaningfully reduces depressive symptoms in adults with clinically diagnosed depression. Given substantial heterogeneity and measurement (self-report vs. observer-rated) and clinical (primary vs. comorbid) variability, any apparent effect modifiers are interpreted cautiously and considered exploratory/hypothesis-generating. To improve precision and implementation, future trials should standardize supervision/adherence reporting (e.g., TIDieR/CERT) and include preregistered follow-ups (3–12 months) to assess durability, while training-prescription guidance remains preliminary pending better-reported, preregistered studies.

**Systematic review registration:**

Unique Identifier: CRD42024583413, https://www.crd.york.ac.uk/PROSPERO/view/CRD42024583413.

## 1 Introduction

Depression affects over 320 million people globally, imposing a substantial burden on individuals and society (Whiteford et al., 2013; Bull et al., 2020). It is a leading cause of years lived with disability worldwide and is associated with elevated risks of various comorbid conditions (e.g., cardiovascular disease, cancer) and other mental disorders such as anxiety (Pinquart and Duberstein, 2010; Rugulies, 2002; Jacobson and Newman, 2017). Although pharmacological and psychotherapeutic treatments are available, many patients struggle with adherence due to side effects (e.g., nausea, insomnia, weight changes, sexual dysfunction) and other challenges, which can diminish overall treatment effectiveness (Zajecka, 2000; Evans-Lacko et al., 2017; Härter et al., 2010). Treatment coverage for depression remains low—fewer than 25% of affected individuals receive adequate care in low- and middle-income countries (versus about 50% in high-income countries) (Moitra et al., 2022). Moreover, depression entails wide-ranging physiological disturbances, impacting metabolic, cardiovascular, hepatic, and immune system function (Tian et al., 2023). These challenges highlight the need for accessible complementary interventions for depression.

Accumulating evidence indicates that exercise is a beneficial adjunct or alternative therapy for depression, with participation conferring improvements in emotional well-being, physical health, and cognitive function (Naci et al., 2019; Hu et al., 2020; Cooney et al., 2013; Singh et al., 2023; Recchia et al., 2022; Owen et al., 2020; Gallardo-Gómez et al., 2022). Large-scale studies have documented high adherence and low adverse event rates for exercise programs in depressed populations, with participation often exceeding 80% (Bull et al., 2020). Consequently, clinical practice guidelines (e.g., the German S3 guideline for unipolar depression) now recommend structured and supervised physical activity as part of standard depression management, emphasizing its feasibility and safety for most patients (Härter et al., 2010).

However, questions remain regarding the optimal exercise modality, intensity, and frequency for treating depression (Zambelli et al., 2022; Malhi et al., 2021; American Psychiatric Association, 2010). Different guidelines reflect this uncertainty: for example, United Kingdom guidelines emphasize group-based activities and encouraging any increase in physical activity (Zambelli et al., 2022; American Psychiatric Association, 2010), whereas the American Psychiatric Association acknowledges both aerobic and resistance exercise as beneficial options (American Psychiatric Association, 2010). The Australian and New Zealand guidelines specifically advise combining strength (resistance) training with high-intensity aerobic exercise at least two to three times per week (Malhi et al., 2021). Likewise, the World Health Organization’s 2020 recommendations for physical activity (150–300 min of moderate or 75–150 min of vigorous aerobic activity per week, plus muscle-strengthening activities on ≥2 days weekly) imply that incorporating RT can yield additional health benefits, including potential mental health gainst (Bull et al., 2020; Tian et al., 2023).

Notwithstanding these recommendations, most research and prior systematic reviews on exercise and depression have focused predominantly on aerobic exercise, whereas RT has received comparatively less attention (Bendau et al., 2022). To date, relatively few meta-analyses have isolated the antidepressant effects of resistance training. For example, Morres et al. reported a large antidepressant effect of endurance (aerobic) exercise (g = −0.79) in adults with major depression (Morres et al., 2019). Initial evidence suggests that RT may produce similarly robust improvements in depressive symptoms (Hirvonen, 2023), but an up-to-date comprehensive synthesis of RCT data focusing specifically on RT is lacking.

Therefore, we conducted a systematic review and meta-analysis of randomized controlled trials to assess the effects of resistance training on depressive symptoms among adults with a clinically diagnosed depressive disorder. To aid interpretation of heterogeneity, moderator evaluations were prespecified as exploratory (with no formal multiplicity correction), including key stratification by clinical phenotype (primary depressive disorder vs. depression secondary to medical comorbidities) and a rater-type sensitivity (self-report vs. observer-rated). All subgroup patterns are interpreted conservatively.

## 2 Methods

### 2.1 Protocol and registration

This review was conducted in accordance with the Preferred Reporting Items for Systematic Reviews and Meta-Analyses (PRISMA) guidelines (Page et al., 2021). The review protocol was registered in the International Prospective Register of Systematic Reviews (PROSPERO; Registration No. CRD42024583413).

### 2.2 Search strategy

A comprehensive literature search was performed in five electronic databases: PubMed, Embase, Web of Science, the Cochrane Central Register of Controlled Trials (CENTRAL), and China National Knowledge Infrastructure (CNKI). The search covered from each database’s inception to 20 August 2024, without language restrictions. We used a combination of Medical Subject Headings (MeSH) and free-text keywords related to “resistance training” (e.g., resistance training, strength training, weightlifting, weight-bearing exercise) and “depression” (e.g., depressive disorder, major depression, depressive symptoms), with filters for human participants and randomized controlled trials. Additionally, the reference lists of all included studies and relevant reviews were screened to identify further eligible trials. To maintain currency during the revision process, we subsequently re-ran the predefined strategy using the same sources and criteria, extending the end date to 10 October 2025; no additional eligible RCTs were identified, and counts were updated in the PRISMA flow. We also searched trial registries (ClinicalTrials.gov, WHO ICTRP, ChiCTR) and grey-literature sources (reference lists of eligible studies and relevant reviews; conference proceedings). The complete PubMed search string is provided in Supplementary Table S1. All records from databases, registries, and grey sources were de-duplicated prior to screening.

### 2.3 Eligibility criteria

Inclusion criteria were defined according to the PICOS framework. Population: Adults (≥18 years) with a clinically diagnosed depressive disorder (DSM/ICD or clinician/psychiatrist diagnosis); trials recruiting solely on elevated depressive-symptom scores were not eligible. Intervention: Structured resistance training (strength training) programs, including exercises using weight machines, free weights, body weight, and/or resistance bands. Comparison: An appropriate non-exercise control condition, such as a wait-list, no-treatment or usual care group, or a control intervention involving minimal physical activity or standard care/psychological therapy without structured exercise. Outcomes: Depressive symptom severity measured by a validated instrument (e.g., Beck Depression Inventory [BDI], Hamilton Rating Scale for Depression [HAM-D or HRSD], Patient Health Questionnaire-9 [PHQ-9], Geriatric Depression Scale [GDS], Depression Anxiety Stress Scales [DASS], Center for Epidemiologic Studies Depression Scale [CES-D], Montgomery–Åsberg Depression Rating Scale [MADRS], Quick Inventory of Depressive Symptomatology [QIDS], etc.). We recorded clinical phenotype (primary depressive disorder vs. depression secondary to medical comorbidities) to enable stratified analyses. Study design: randomized controlled trials.

Studies were excluded if they were animal experiments, lacked an appropriate control group (or used an active exercise control that precluded isolating RT effects), or were published only as abstracts, letters/commentaries, or other formats without original data (e.g., narrative reviews or conference proceedings without extractable trial data). Duplicate publications and secondary analyses were excluded to avoid overlapping data.

### 2.4 Study selection

All records identified from databases, trial registries, and grey sources were imported into a reference manager, and duplicates were removed. Two reviewers independently screened the titles and abstracts of the remaining records and excluded obviously irrelevant studies. Full-text articles of potentially relevant studies were then retrieved and assessed against the inclusion criteria. Any discrepancies or disagreements in study selection were resolved through discussion, with consultation of a third reviewer if needed.

For each study meeting the inclusion criteria, two reviewers independently extracted key data using a standardized form. The following information was collected: authors, publication year, country, sample size and characteristics (age, sex distribution, clinical population or any comorbid conditions), clinical phenotype (primary depressive disorder vs. depression secondary to medical comorbidities), details of the RT intervention (type of exercises, equipment used, intensity, session duration, frequency per week, total intervention length, supervision), details of the control condition, outcome measures for depression (specific scales used), and main results (mean depression scores at baseline and post-intervention for each group, or mean changes, along with standard deviations [SDs]). When multiple depression instruments were reported at the same time point, we selected a single prespecified primary outcome per trial using a hierarchy (protocol-defined primary > observer-rated > self-report) to avoid double counting. We also recorded adherence (attendance rates to training sessions) and any reported adverse events or drop-outs.

For continuous outcomes, we extracted means and SDs of depression scores at post-intervention (or change-from-baseline scores when provided) for the RT and control groups. If a study did not report the necessary summary data, we contacted the authors to request the information. When data remained unavailable, we employed standard estimation methods to derive missing values. Specifically, if a post-intervention mean was not reported but baseline and change scores were available, we calculated the post-intervention mean as baseline plus change. If a post-intervention SD was not reported, we imputed it using established formulas. In one approach, we assumed a moderate correlation (Corr ≈ 0.524) between baseline and post-test measures and derived the missing SD accordingly (Gates et al., 2013). For small samples (e.g., *n* < 60), SDs were estimated from reported confidence intervals using a t-distribution approach (Chen et al., 2020). For studies that reported multiple follow-up time points, only the final assessment (closest to the end of the intervention period) was used in our analysis to represent the post-intervention outcome.

Subgroup classifications were prespecified to aid interpretation of heterogeneity and were treated as exploratory without formal multiplicity correction; strata with k < 5 were flagged as imprecise. Age was categorized as young (<30 years), middle-aged (30–60 years), or elderly (>60 years). Depression severity was classified as mild, moderate, or severe according to clinical definitions or scale cut-offs (for example, BDI scores of 14–19 indicating mild, 20–28 moderate, and ≥29 severe depression). RT program length was defined as short-term (4–8 weeks), medium-term (9–24 weeks), or long-term (≥24 weeks) (Chen et al., 2020). Training frequency was the primary prescription moderator and was coded as <3 vs. ≥ 3 sessions per week for analysis (Chen et al., 2020). Training intensity was categorized as low (≤50% of one-repetition maximum [1RM]), moderate (50–75% 1RM), or high (≥75% 1RM) (Norton et al., 2010). Weekly training volume (duration) was classified as <120 min per week, 120–180 min per week, or >180 min per week (Northey et al., 2018).

### 2.5 Risk of bias assessment

Two reviewers independently evaluated the risk of bias of each included RCT using the Cochrane Risk of Bias tool (Higgins et al., 2011). The following domains were assessed: sequence generation (randomization), allocation concealment, blinding of participants and personnel, blinding of outcome assessment, completeness of outcome data, selective outcome reporting, and other potential sources of bias. Each domain was rated as “low risk,” “high risk,” or “unclear risk” of bias according to Cochrane Handbook criteria. Discrepancies between reviewers were resolved through discussion or by involving a third reviewer. We used the overall risk-of-bias assessments to inform the interpretation of the findings, considering how any methodological limitations might affect confidence in the results.

### 2.6 Data analysis

We used Hedges’ g (small-sample–corrected) standardized mean differences (SMDs) with 95% confidence intervals to quantify the effect of RT on depressive symptom scores, since the studies used a variety of depression measurement scales. For each trial, we computed the SMD between the RT intervention group and the control group at post-intervention. These SMDs were pooled using a random-effects model (DerSimonian-Laird method) to account for expected variability in populations and interventions across studies. In multi-arm trials, unit-of-analysis errors were avoided by combining intervention arms or by splitting shared controls as described above.

Statistical heterogeneity was evaluated with Cochran’s Q test and quantified by the *I*^2^ statistic (Kampshoff et al., 2015; Palesh et al., 2018). An *I*^2^ value greater than 50% was considered indicative of substantial heterogeneity in effects between studies. Moderator evaluations were prespecified as exploratory (no formal multiplicity correction), and strata with k < 5 were flagged as imprecise. The primary prescription moderator was training frequency (<3 vs. ≥ 3 sessions/week); key clinical stratification contrasted primary depressive disorder versus depression secondary to medical comorbidities; a rater-type sensitivity (self-report vs. observer-rated) was also planned. We explored heterogeneity using prespecified, exploratory moderator analyses and random-effects meta-regression. The key moderators were clinical phenotype (primary depressive disorder vs. depression secondary to medical comorbidities) and training frequency (<3 vs. ≥ 3 sessions/week). Additional exploratory moderators included age, baseline severity, intervention duration, training intensity, and weekly training volume. Subgroup syntheses used random-effects models; no formal multiplicity correction was applied and strata with k < 5 were flagged as imprecise. We also conducted a rater-type sensitivity (self-report vs. observer-rated) to address measurement heterogeneity.

We assessed publication bias by inspecting a funnel plot of the pooled outcome for asymmetry and by conducting Begg’s rank correlation test for small-study effects. Given the study count and between-study heterogeneity, we interpreted these tests with caution due to limited power. In addition, we carried out a sensitivity analysis to test the robustness of the results: (i) exclusion of studies at high risk of bias (quality-informed sensitivity) and (ii) leave-one-out analyses to determine whether any single trial was unduly influencing the overall effect estimate. All statistical analyses were performed using Review Manager (RevMan) 5.4 and Stata version 16.0. Because this study synthesized data from previously published studies and did not involve any new collection of human subject data, separate ethical approval was not required.

## 3 Results

### 3.1 Study selection

**Figure 1:**
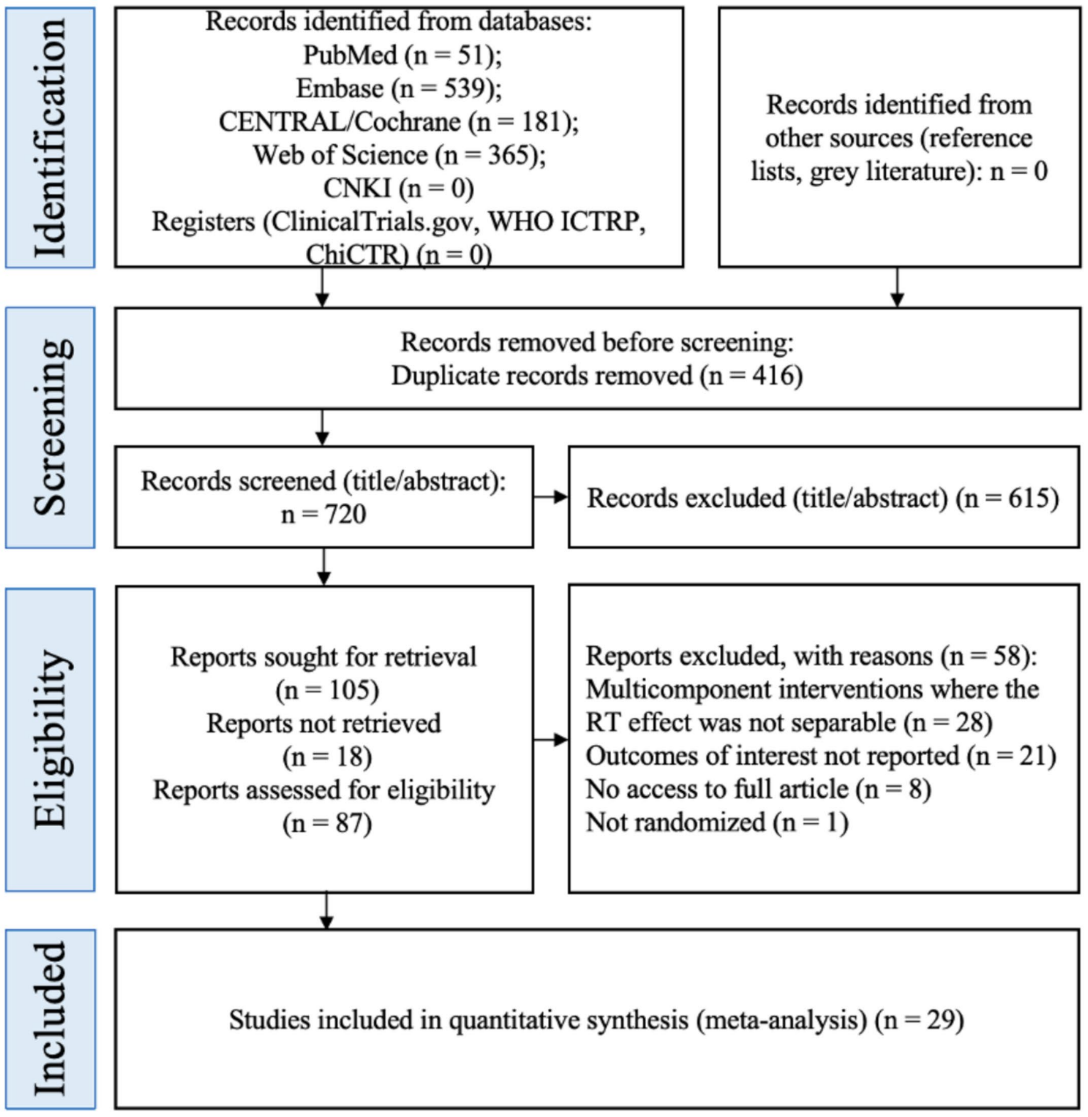
PRISMA flowchart.

The database search yielded 1,136 records (Figure 1): PubMed (*n* = 51), Embase (*n* = 539), Cochrane CENTRAL (*n* = 181), Web of Science (*n* = 365), and CNKI (*n* = 0). In addition, we searched trial registries (ClinicalTrials.gov, WHO ICTRP, ChiCTR) and grey-literature sources; no additional records were identified from these sources. After removing 416 duplicates, 720 records remained for title/abstract screening, of which 615 were excluded as irrelevant. We sought full texts for 105 reports; 18 could not be retrieved. The remaining 87 reports were assessed for eligibility, and 58 were excluded with reasons (multicomponent interventions where the RT effect was not separable, *n* = 28; outcomes of multicomponent interventions where the RT effect was not separable, *n* = 28; outcomes of interest not reported, *n* = 21; no access to full article, *n* = 8; not randomized, *n* = 1). Ultimately, 29 randomized controlled trials were included in the quantitative synthesis (meta-analysis).

### 3.2 Trial characteristics

A total of 29 RCTs, encompassing 2,036 participants with depression, were included in this meta-analysis. Key characteristics of the included trials are summarized in Table 1. Depression severity: Of the total participants, 281 were classified as having mild depression, 1,047 as moderate depression, and 708 as severe depression at baseline. Group allocation: Across all studies, 1,028 individuals were assigned to RT intervention groups and 1,008 to control groups. Geographic distribution: The trials were conducted in a wide range of countries. The United States contributed the most studies (9 trials), followed by Brazil (4 trials), Australia (3), China (2), Finland (2), and Turkey (2). Additionally, one trial each was conducted in Denmark, Germany, Belgium, Japan, Canada, Ireland, and Switzerland. All of the included studies were published in English.

**Table 1:** Characteristics of the included studies.

Study	Country	Region	Number (Female/Men)	Mean age (SD/Range)	Comorbidities	Baseline depression	Type of exercise	Intensity	Intervention program (RT: high: >75% 1RM, moderate: 50–75% 1RM, low: <50% 1RM)	Length (weeks)	Frequency (times/time)	Volume (min/week)	Supervised or nonsupervised	Outcomes measured
Sun et al. (2022)	China	Asian	145 (118/27)	33.1 ± 5.9	Vestibular migraine	Moderate	RT	Moderate	Resistance exercise session lasted 60 min and at the weight of 60–80% 1RM	12	2/60	120	Supervised	BDI
			141 (117/24)	34.5 ± 6.3	Vestibular migraine	Moderate	CON		Patients in the relaxation control group were instructed to perform gradual muscle relaxations					BDI
Doyne et al. (1987)	USA	North America	30 (30/0)	27.67 ± 4.81	NR	Moderate	RT	NR	Subjects used a Universal Exercise Machine and went through a standard 10-station program	8	4/NR	NR	Supervised	BDI
			30 (30/0)	29.46 ± 4.68		Moderate	CON		Wait-list control					BDI
Singh et al. (2005)	USA	North America	20 (11/9)	69 ± 5	NR	Severe	RT	Vigorous	Exercise machines included chest press, upright row, shoulder press, leg press, knee extension, and knee flexion. Resistance was set at 80% of the one repetition maximum	8	3/60	180	Supervised	HRSD GDS
			20 (10/10)	69 ± 7	NR	Severe	CON		A usual care					HRSD GDS
Singh et al. (2005)	USA	North America	20 (12/8)	70 ± 7	NR	Severe	RT	Low	Exercise machines included chest press, upright row, shoulder press, leg press, knee extension, and knee flexion. The resistance was set at 20% of the one repetition maximum	8	3/60	180	Supervised	HRSD GDS
			20 (10/10)	69 ± 7	NR	Severe	CON		A usual care					HRSD GDS
Zhao et al. (2023)	China	Asian	29 (20/9)	21.66 ± 1.97	NR	Moderate	RT	Vigorous	RT was conducted with dumbbells and elastic bands.	12	3/35	105	Supervised	SDS
			28 (20/8)	21.21 ± 2.25	NR	Moderate	CON		Wait list					SDS
Sims et al. (2009)	Australia	Oceania	23 (9/14)	67.95 ± 14.76	Stroke	Severe	RT	Vigorous	The core PRT program entailed intensity (three sets of eight/ten repetitions, at a resistance of 80% of 1-RM)	10	2/NR	NR	Supervised	CES-D
			22 (9/13)	66.27 ± 16.01	Stroke	Severe	CON		A usual care				Supervised	CES-D
Deus et al. (2021)	Brazil	South America	81 (46/35)	67.27 ± 3.24	Hemodialysis	Severe	RT	NR	Patients completed twelve exercises each session which takes ~60 min. They conducted three sets of 8-to-12 repetitions	24	3/60	180	Supervised	BDI
			76 (40/36)	66.33 ± 3.88	Hemodialysis	Severe	CON		A usual care					BDI
Krogh et al. (2007)	Denmark	Europe	55 (45/10)	41.9 ± 8.7	NR	Severe	RT	Moderate	The training is a circle-training program involving large muscle groups including both machines and free weights	4 (months)	2/60	120	Supervised	BDI, HAM-D, MADRS
			55 (34/21)	36.7 ± 8.7	NR	Severe	CON		Main exercises are done at an intensity level at 6–10 on the Borg scale					BDI, HAM-D, MADRS
Makizako et al. (2019)	Japan	Asian	30 (16/14)	73.1 ± 5.3	NR	Moderate	RT	NR	20 min of muscle strength exercises and postural balance re-training	24	20/90	1800	Supervised	GDS-15
			29 (15/14)	73.0 ± 5.9	NR	Moderate	CON		Two 90-min education classes					GDS-15
Singh et al. (2001)	USA	North America	17 (11/6)	71 ± 2.0	NR	Moderate	RT	Vigorous	3 days a week for 10 weeks. The resistance was set at 80% of the one-repetition maximum (1-RM), and subjects performed three sets of eight repetitions	20	3/45	135	Supervised-nonsupervised	BDI
			15 (9/6)	71 ± 2.0	NR	Moderate	CON		Education classes					BDI
Moraes et al. (2020)	Brazil	South America	9 (8/1)	72.89 ± 7.06	NR	Moderate	RT	Moderate	70% of 1RM, 3sets, 8-12reps	12	2/30	60	Supervised	BDI HAM-D
			7 (5/2)	69.28 ± 5.28	NR	Moderate	CON		Low-intensity exercise					BDI HAM-D
Luttenberger et al. (2015)	Germany	Europe	22 (12/10)	42.71 ± 11.88	NR	Moderate	RT	NR	Bouldering therapy	8	1/180	180	Supervised	BDI-II
			25 (15/10)	44.96 ± 12.08	NR	Moderate	CON		Wait list					BDI-II
Ciccolo et al. (2022)	USA	North America	25 (0/25)	41.68 ± 13.44	NR	Moderate	RT	Moderate	40 min of RT at 50–70% 1RM	12	2/60	120	Supervised	PHQ-9
			25 (0/25)	41.00 ± 11.29	NR	Moderate	CON		Education classes					PHQ-9
Abrahão et al. (2016)	Brazil	South America	21(NR)	39.1 ± 14.4	Lupus erythematosus	Moderate	RT	Moderate	Freeweight and elastic band exercises for 50 min, three times a week	12	3/50	150	Supervised	BDI
			21(NR)	46.1 ± 14.1	Lupus erythematosus	Moderate	CON		A usual care					BDI
Vizza et al. (2016)	Australia	Oceania	7 (7/0)	26 ± 7	Polycystic ovary syndrome	Moderate	RT	Moderate	11 PRT exercises	12	3/60	180	Supervised	DASS
			6 (6/0)	29 ± 3	Polycystic ovary syndrome	Moderate	CON		A usual care					DASS
Sparrow et al. (2011)	USA	North America	52(NR)	70.3 ± 7.5	NR	Mild	RT	Vigorous	8 resistance exercises	12 (months)	3/60	180	Supervised	BDI
			51(NR)	71.7 ± 7.2	NR	Mild	CON		General health education					BDI
Sabapathy et al. (2011)	Australia	Oceania	16 (12/4)	55 ± 7	Multiple sclerosis	Mild	RT	Moderate	Three upper body and three lower body exercises as well as one core strength and one stability exercise	8	2/60	120	Supervised	BDI
			16 (12/4)	55 ± 7	Multiple sclerosis	Mild	CON		Low-intensity exercise					BDI
Redwine et al. (2020)	USA	North America	22 (3/19)	65 ± 9	Heart failure	Mild	RT	Low	Resistance band	16	2/60	120	Supervised	BDI
			23 (3/20)	67 ± 7	Heart failure	Mild	CON		treatment as usual					BDI
Piraux et al. (2021)	Belgium	Europe	24 (0/24)	67.9 ± 7.1	Prostate cancer	Mild	RT	Low	Resistance program consisted of eight exercises sets of 8–12 repetitions	8	3/40	120	Supervised	CES-D
			24 (0/24)	71.9 ± 8.1	Prostate cancer	Mild	CON		General health education					CES-D
Palmer et al. (1995)	USA	North America	15(NR)	18–55	NR	Severe	RT	Moderate	Subjects in the bodybuilding group performed three sets of the 10 exercises	4	3/35	105	Supervised	CES-D
			15(NR)	18–55	NR	Severe	CON		Aerobic step					CES-D
Kekäläinen et al. (2018)	Finland	Europe	28 (16/12)	69.0 ± 3.3	NR	Mild	RT	low	Each training session lasted for 1 h, 8–9 exercises for different muscle groups	3 (months)	3/60	180	Supervised	BDI-II
			23 (11/12)	68.3 ± 2.3	NR	Mild	CON		No exercise					BDI-II
Herring et al. (2011)	USA	North America	10 (10/0)	18–37	NR	Moderate	RT	Moderate	seven sets of 10 repetitions each of leg press, leg curl and leg extension exercises	6	2/NR	NR	Supervised	BDI-II
			10 (10/0)	18–37	NR	Moderate	CON		Wait list					BDI-II
HÃ¤kkinen et al. (2001)	Finland	Europe	11 (11/0)	39 ± 6	Fibromyalgia	Mild	RT	Moderate	Training was carried out twice a week and each training session included six to eight exercises	21	2/NR	NR	Supervised	BDI
			10 (10/0)	37 ± 5	Fibromyalgia	Mild	CON	NR	No exercise	21				BDI
De Lima et al. (2019)	Brazil	South America	17(NR)	66.2 ± 5.5	Parkinson	Moderate	RT	NR	Two sets of 8–12 repetitions of each of the following exercises	20	2/30–40	60–80	Supervised	HRSD
			16(NR)	67.2 ± 5.2	Parkinson	Moderate	CON	NR	No exercise	20				HRSD
Courneya et al. (2007)	Canada	North America	82 (82/0)	≥18	Breast Cancer	Moderate	RT	Moderate	Two sets of 8 to 12 repetitions of nine different exercises	6 (months)	3/45	135	Supervised	CES-D
			82 (82/0)	≥18	Breast Cancer	Moderate	CON	NR	No exercise					CES-D
Bosnak-Guclu et al. (2011)	Turkey	Asian	16 (12/4)	69.50 ± 7.96	Heart failure	Mild	RT	Low	Inspiratory muscle training	6	7\30	210	Supervised-nonsupervised	MADRS
			12 (12/2)	65.71 ± 10.52	Heart failure	Mild	CON		Inspiratory muscle training-free of pressure					MADRS
Çetinkaya and Karakoyun (2022)	Turkey	Asian	30 (27/3)	60–65 (10) 66–70 (15) ≥71 (5)	NR	Moderate	RT	NR	The elastic band exercises	4	NR	NR	Supervised-nonsupervised	BDI
			30 (26/4)	60–65 (12) 66–70 (12) ≥71 (6)		Moderate	CON		No exercise					BDI
O’Sullivan et al. (2023)	Ireland	Europe	26 (17/9)	25.8 ± 5.7	NR	Moderate	RT	Moderate	8 resistance exercises	8	2\25	50	Supervised	QIDS
			29 (18/11)	27.5 ± 5.7	NR	Moderate	CON		No exercise					QIDS
Eisenhut et al. (2022)	Switzerland	Europe	11 (11/0)	54.6 ± 13.45	High-grade glioma	Moderate	RT	Moderate	Perform weightlifting and resistance exercises with proper equipment.	6	2/35–45	75–90	Supervised	BDI
			8 (8/0)	53.0 ± 10.78	High-grade glioma	Moderate	CON		No exercise					BDI

RT: Resistance Training; BDI (Beck Depression Inventory), BDI-II (Revised BDI), GDS (Geriatric Depression Scale), CES-D (Center for Epidemiologic Studies Depression Scale), HADS (Hospital Anxiety and Depression Scale, Depression Subscale), Montgomery– (Åsberg Depression Rating Scale) PHQ-9 (Patient Health Questionnaire-9), CSDD (Cornell Scale for Depression in Dementia), CGI (Clinical Global Impression Scale, Depression Subscale), CDRS (Children’s Depression Rating Scale), QIDS (Quick Inventory of Depressive Symptomatology); NR: Not Reported.

#### 3.2.1 Participant and clinical profiles

All trials enrolled participants with a clinical diagnosis of depressive disorder (DSM/ICD or clinician/psychiatrist diagnosis), ensuring a well-defined clinical sample. The majority of studies focused on adults under 65, though a few included older adults (up to 75 years). Thirteen trials (combined *n* = 908) included participants with notable medical comorbidities such as stroke, Parkinson’s disease, chronic kidney disease requiring hemodialysis, heart failure, or breast cancer; in these studies, depression was often a comorbid condition alongside the primary diagnosis. Adherence and safety reporting varied: fourteen studies (48.3%) reported participants’ adherence to the exercise sessions, with attendance rates ranging from approximately 50 to 96% of scheduled sessions (mean attendance about 80%). Adverse events related to the interventions were documented in 14 studies (common issues were transient muscle soreness or fatigue, with no serious exercise-related adverse events reported), while 9 studies explicitly stated that no adverse events occurred.

#### 3.2.2 Intervention details

The resistance training programs varied in format and intensity, but several patterns emerged. Training frequency ranged from 1 session per week to daily sessions (7 per week), with most trials (24 out of 29) implementing a moderate frequency of 2–3 sessions per week. Session durations also varied widely, from ~25 min up to 180 min per session; however, the majority of interventions used 30–60 min training sessions. The total intervention duration (program length) ranged from 4 weeks to 48 weeks. The most common intervention lengths were 8 weeks (8 studies) and 12 weeks (6 studies), while a few studies ran longer programs of 6 months or more. Resistance training intensity was typically described in terms of percentage of one-repetition maximum (1RM) or as low/moderate/vigorous; many interventions in the included trials targeted moderate to high intensity (around 60–80% of 1RM), although one trial explicitly tested a low-intensity regimen for comparison. Most studies employed supervised RT protocols (with exercise physiologists or trainers overseeing sessions), except a few that allowed unsupervised home-based training with periodic check-ins.

#### 3.2.3 Depression outcome measures

Both self-report inventories (e.g., BDI/BDI-II, CES-D, PHQ-9, DASS, SDS, QIDS) and observer-rated scales (e.g., HAM-D/HRSD, MADRS; GDS in older adults) were used across the studies. The most frequently used assessment was the Beck Depression Inventory (BDI) in 12 studies (with an additional 3 studies using the BDI-II revision). Four trials used the Center for Epidemiologic Studies Depression Scale (CES-D). Three trials employed the Hamilton Depression Rating Scale (HAM-D or HRSD), and three used the Geriatric Depression Scale (GDS) for older participants. Two studies measured depression with the Hospital Anxiety and Depression Scale (HADS-D, depression subscale), two with the Montgomery–Åsberg Depression Rating Scale (MADRS), and one study each used the Self-Rating Depression Scale (SDS), the Patient Health Questionnaire-9 (PHQ-9), the Depression subscale of the Depression Anxiety Stress Scales (DASS), and the Quick Inventory of Depressive Symptomatology (QIDS). Despite this variety of scales, all studies provided a quantitative measure of depressive symptom severity before and after the intervention, enabling the calculation of effect sizes (Table 1 provides further details on each study’s design and outcomes).

### 3.3 Risk of bias

**Figure 2:**
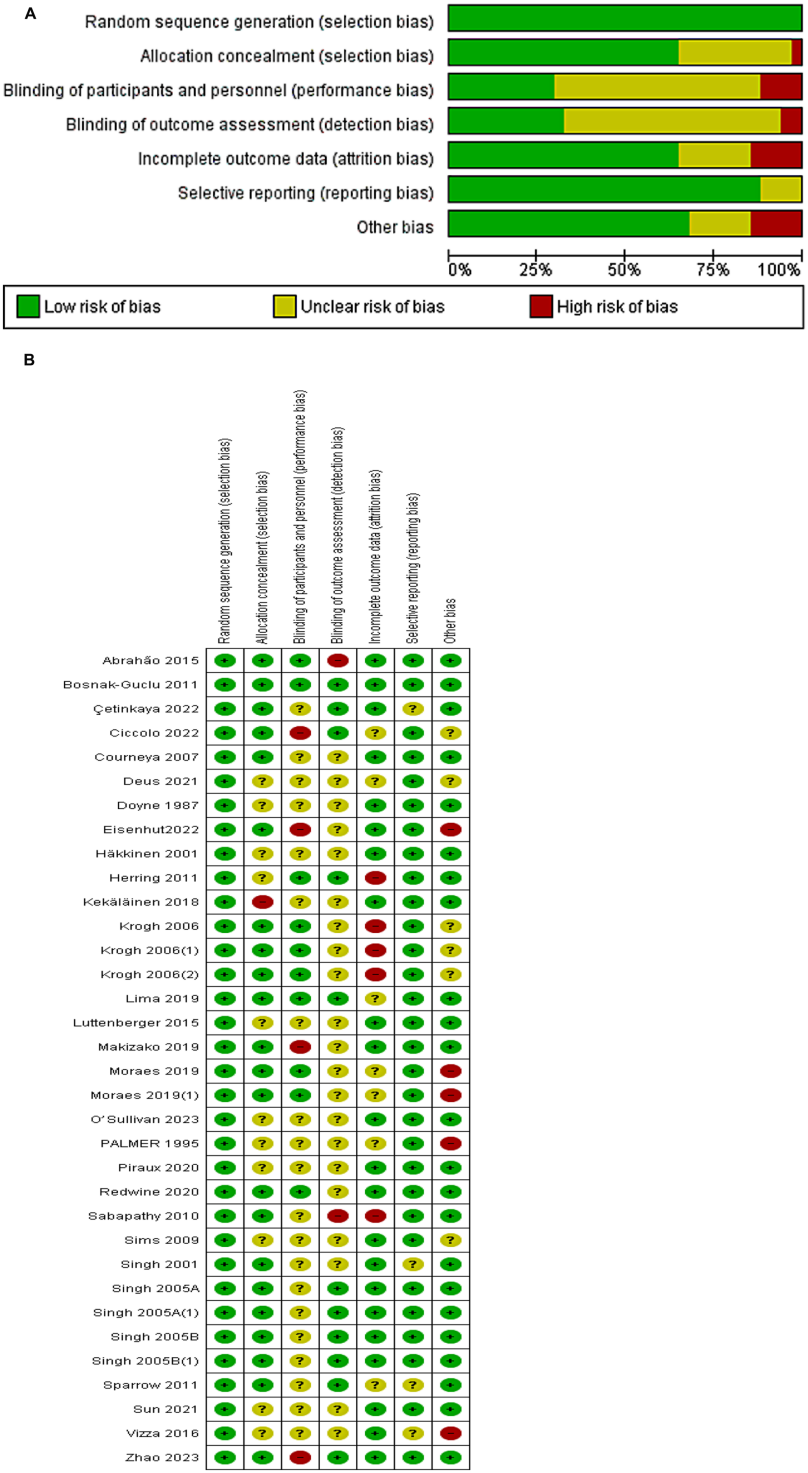
**(A)** Risk of bias of each study. **(B)** Risk of bias summary.

Overall risk of bias was moderate across the included studies, with some methodological limitations noted (Figures 2A,B summarize the risk of bias assessments). All 29 trials stated that participants were randomly assigned to groups, and 17 of these provided clear descriptions of the allocation concealment methods (such as use of sealed opaque envelopes or centralized randomization) to prevent selection bias. The blinding of participants and personnel was generally not feasible due to the nature of exercise interventions – indeed, most studies did not report any blinding in this domain, reflecting the inherent difficulty of blinding subjects to an exercise vs. no-exercise condition. This lack of blinding could introduce some performance bias (participants’ and instructors’ awareness of the intervention might influence outcomes or behavior). Blinding of outcome assessors was reported in 9 studies, helping to mitigate detection bias in those cases; the remaining studies either did not specify assessor blinding or used self-reported outcomes only.

Attrition bias was low in most trials: 20 studies were rated as low risk for incomplete outcome data. Eighteen trials had no or minimal dropout, while others provided acceptable handling of missing data (e.g., last observation carried forward or intention-to-treat analyses). Selective reporting bias was also largely low; 25 trials clearly reported the depression outcomes prespecified in their methods or protocols. Seven studies had other potential sources of bias marked as unclear or high risk (e.g., small sample sizes, baseline imbalances, or limited detail on intervention fidelity). Only one trial met the criterion for overall low risk of bias; therefore, a formal “low vs. non-low” subgroup comparison was not informative. Quality-informed sensitivity analyses are reported in Section 3.6: excluding high-risk trials yielded a pooled effect similar in magnitude to the main analysis, and leave-one-out checks indicated no single study unduly influenced the results. In summary, while certain biases—particularly the infeasibility of participant/personnel blinding—are unavoidable, the included RCTs were generally of reasonable methodological quality and their results can be considered with moderate confidence.

### 3.4 Overall effect of resistance training on depression

**Figure 3:**
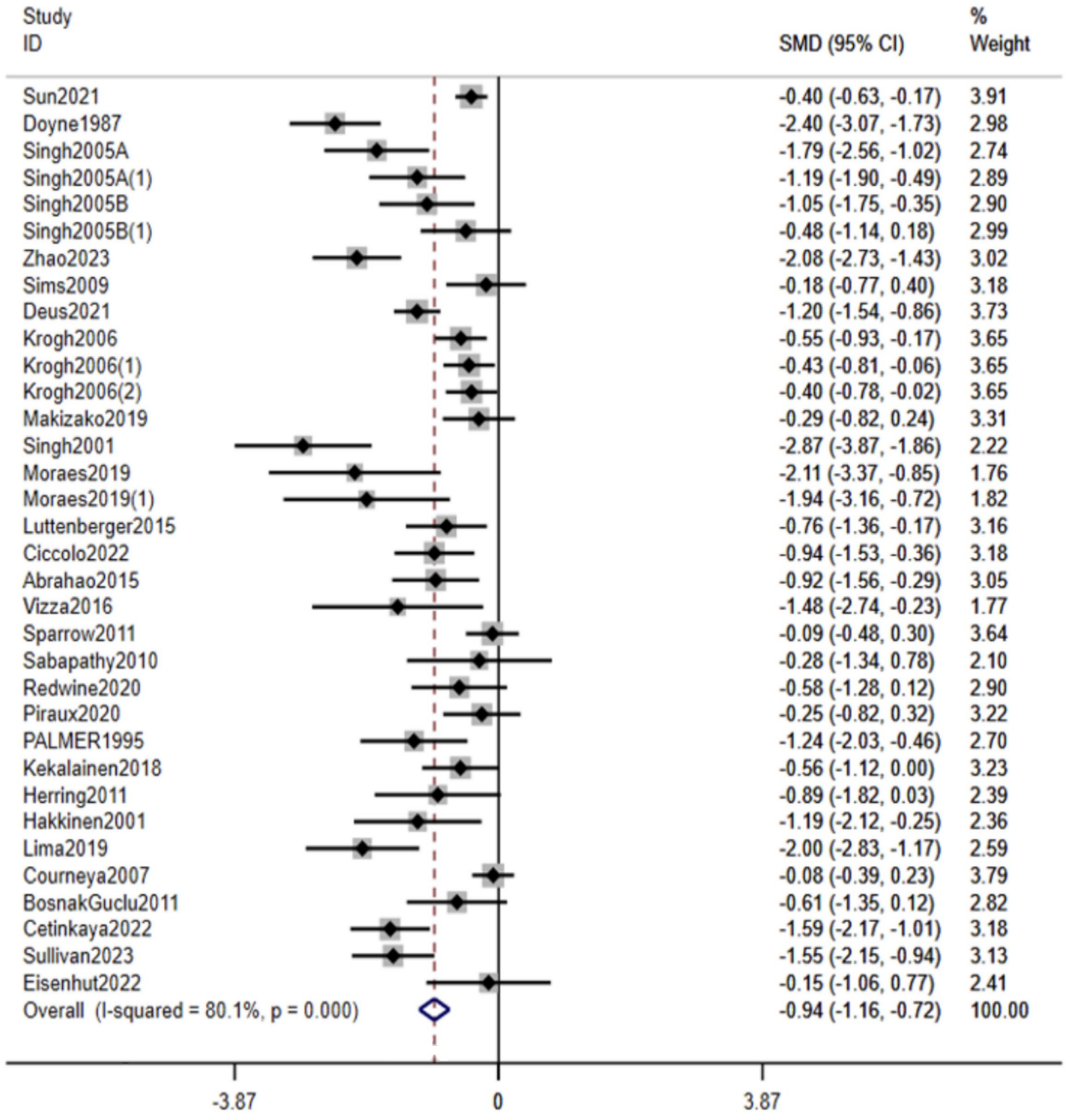
Forest plot of RT’s effect on depression. Weights are from random effects analysis.

Combining the data from all 29 RCTs (2,036 total participants), we found that resistance training had a significant antidepressant effect compared to no exercise or usual care controls. The random-effects meta-analysis yielded a pooled SMD of −0.94 (95% CI: −1.16 to −0.72, *p* < 0.001) in favor of the RT intervention (Figure 3). Given that SMDs are scale-free and studies used different instruments, we describe this as a moderate improvement and interpret the magnitude cautiously in light of heterogeneity. There was, however, substantial heterogeneity in effect sizes across studies (Q-test *p* < 0.001, *I*^2^ = 80.1%), indicating that the magnitude of RT’s benefits varied considerably between trials. Directionally, most studies favored RT, and quality-informed sensitivity analyses yielded estimates of similar magnitude, supporting robustness. Overall, these results demonstrate that RT, on average, produces a markedly greater improvement in depressive symptoms than control conditions, reinforcing the value of resistance exercise as an intervention for depression.

### 3.5 Subgroup analysis

To explore the observed heterogeneity and gain insight into conditions under which RT is most effective, we conducted subgroup analyses based on study and participant characteristics and intervention features. All subgroup and moderator evaluations were prespecified as exploratory, and no formal multiplicity correction was applied; therefore, inferences are conservative. Subgroups with small study counts (e.g., k < 5) are flagged as imprecise, and any apparent differences are regarded as hypothesis-generating rather than confirmatory.

#### 3.5.1 Clinical phenotype

**Figure 4:**
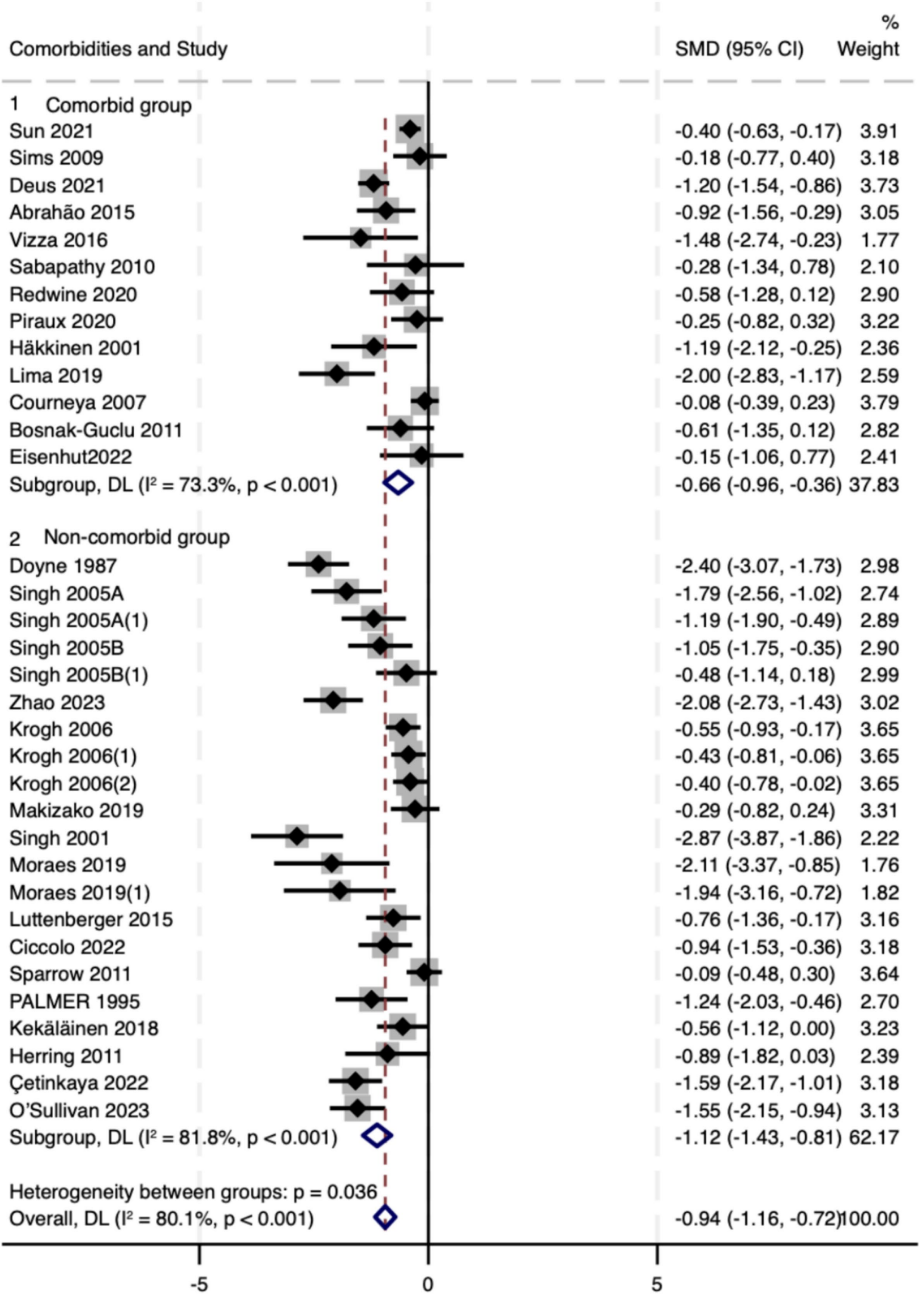
Forest plot of subgroup analyses stratified by clinical phenotype. Weights and between subgroup heterogeneity test are from random-effects model.

When stratified by clinical phenotype, resistance training was associated with reductions in depressive symptoms in both strata: comorbid depression (*k* = 13; SMD − 0.66, 95% CI − 0.96 to −0.36; *I*^2^ = 73.3%) and primary depressive disorder (*k* = 21; SMD − 1.12, −1.43 to −0.81; *I*^2^ = 81.8%). A modest between-subgroup contrast favored primary depression (Q_between = 4.41, *p* = 0.036; Figure 4). Meta-regression provided a concordant signal of attenuation in comorbid samples (ratio of effects ≈0.65; Knapp–Hartung *p* = 0.085; Adj. R^2^ = 6.6%). Taken together, these findings indicate benefit across phenotypes, with somewhat smaller effects in comorbid populations; estimates should be interpreted cautiously given substantial within-stratum heterogeneity and the exploratory nature of subgrouping.

#### 3.5.2 Intervention frequency

**Figure 5:**
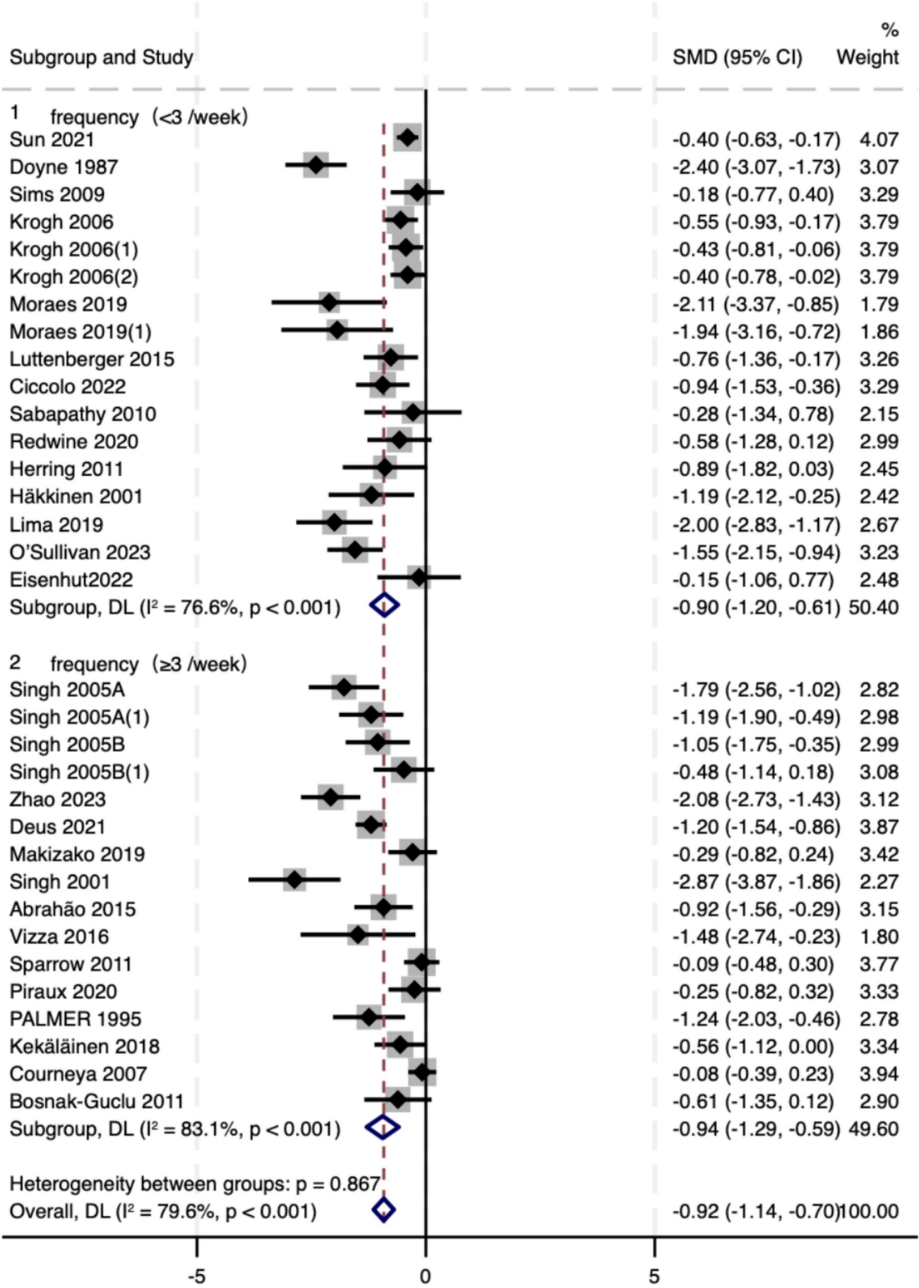
Forest plot of subgroup analyses stratified by intervention frequency. Weights and between subgroup heterogeneity test are from random-effects model.

Training frequency was prespecified as the primary prescription moderator and analyzed dichotomously (<3 vs. ≥ 3 sessions/week; Figure 5). Effects were directionally consistent across strata: <3 sessions/week: SMD = −0.90 (95% CI − 1.20 to −0.61; *I*^2^ = 76.6%) and ≥3 sessions/week: SMD = −0.94 (95% CI − 1.29 to −0.59; *I*^2^ = 83.1%). The between-subgroup contrast was not statistically compelling (Q_between *p* = 0.867). One study had unreported frequency (NR); excluding this NR trial did not materially change the overall estimate (overall SMD ≈ −0.92, 95% CI − 1.14 to −0.70). Taken together, these results indicate benefit across commonly used frequencies without evidence for a clearly superior category; inferences remain exploratory.

#### 3.5.3 Rater type

**Figure 6:**
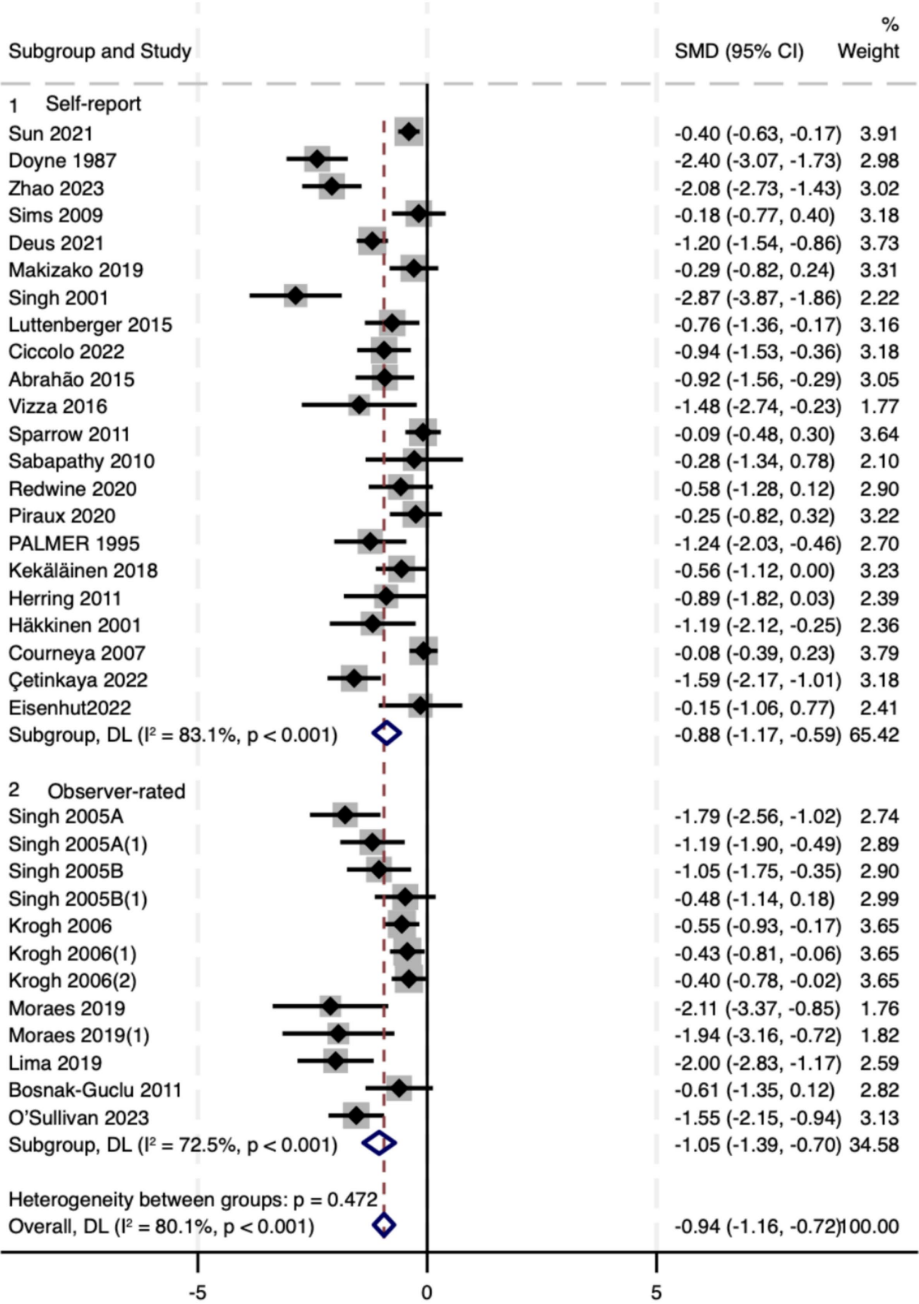
Forest plot of subgroup analyses stratified by rater type. Weights and between subgroup heterogeneity test are from random-effects model.

Effects were directionally consistent across measurement approaches. Pooled estimates were Self-report: SMD − 0.88 (95% CI − 1.17 to −0.59) and Observer-rated: SMD − 1.05 (95% CI − 1.39 to −0.73), with no compelling between-subgroup difference (Q_between = 0.47, *p* = 0.472; Figure 6). Given that this analysis was prespecified as exploratory and that rater types may capture partially distinct constructs of depression, we interpret these findings conservatively and view any apparent differences as hypothesis-generating.

#### 3.5.4 Age

**Figure 7:**
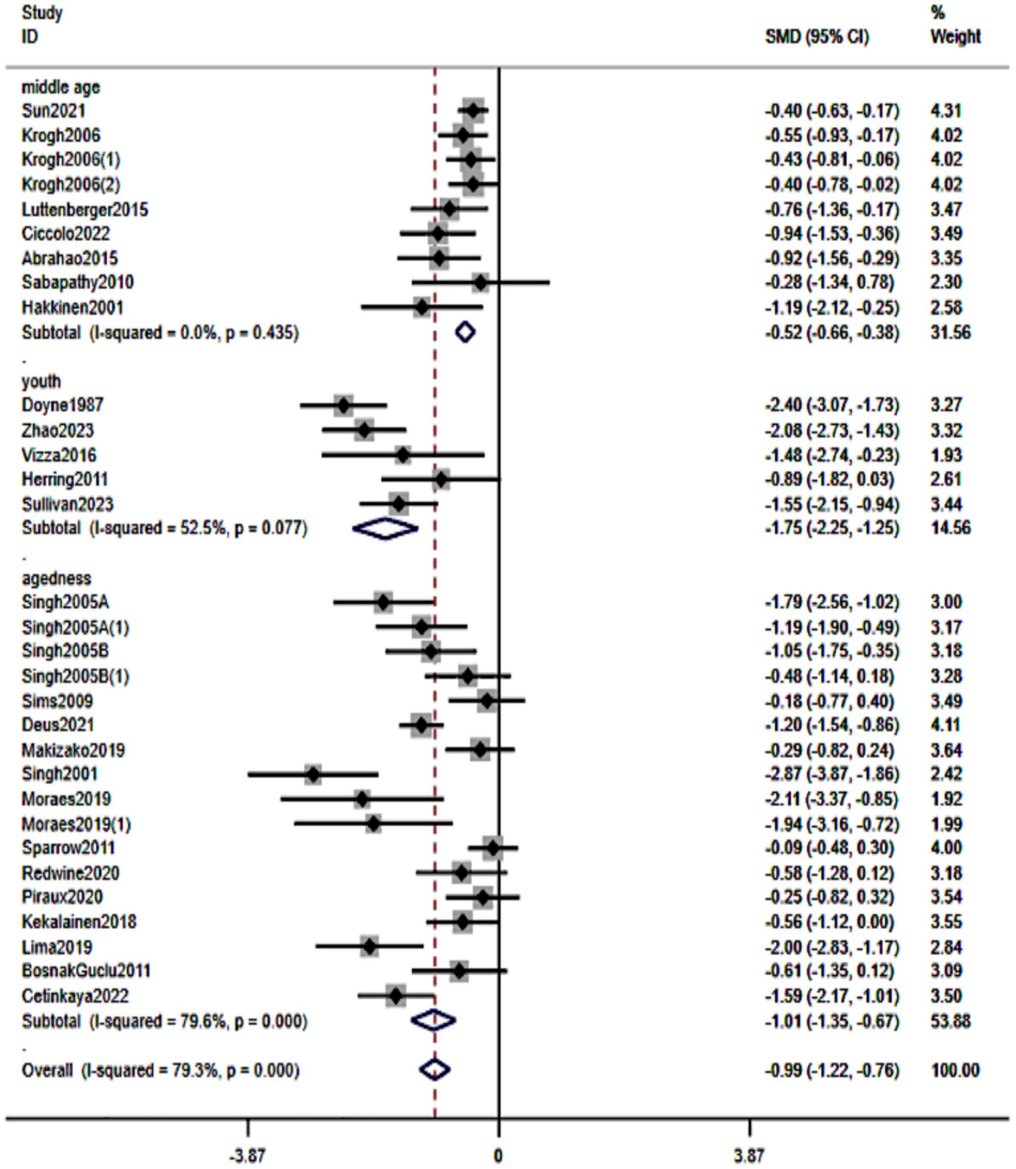
Forest plot of subgroup analyses stratified by age. Weights are from random effects analysis.

When stratified by age (Figure 7), resistance training was associated with reduced depressive symptoms across the adult lifespan. Pooled effects were −1.75 (95% CI − 2.25 to −1.25) in younger adults (≈18–29 y; *n* = 205), −0.52 (−0.66 to −0.38) in middle-aged adults (≈30–60 y; *n* = 1,021), and −1.01 (−1.35 to −0.67) in older adults (>60 y; *n* = 810). Although point estimates were larger in younger samples, confidence intervals overlapped and the smallest stratum yielded imprecise estimates. Overall, these findings indicate benefits across age groups without compelling evidence for age-related superiority.

#### 3.5.5 Depression severity

**Figure 8:**
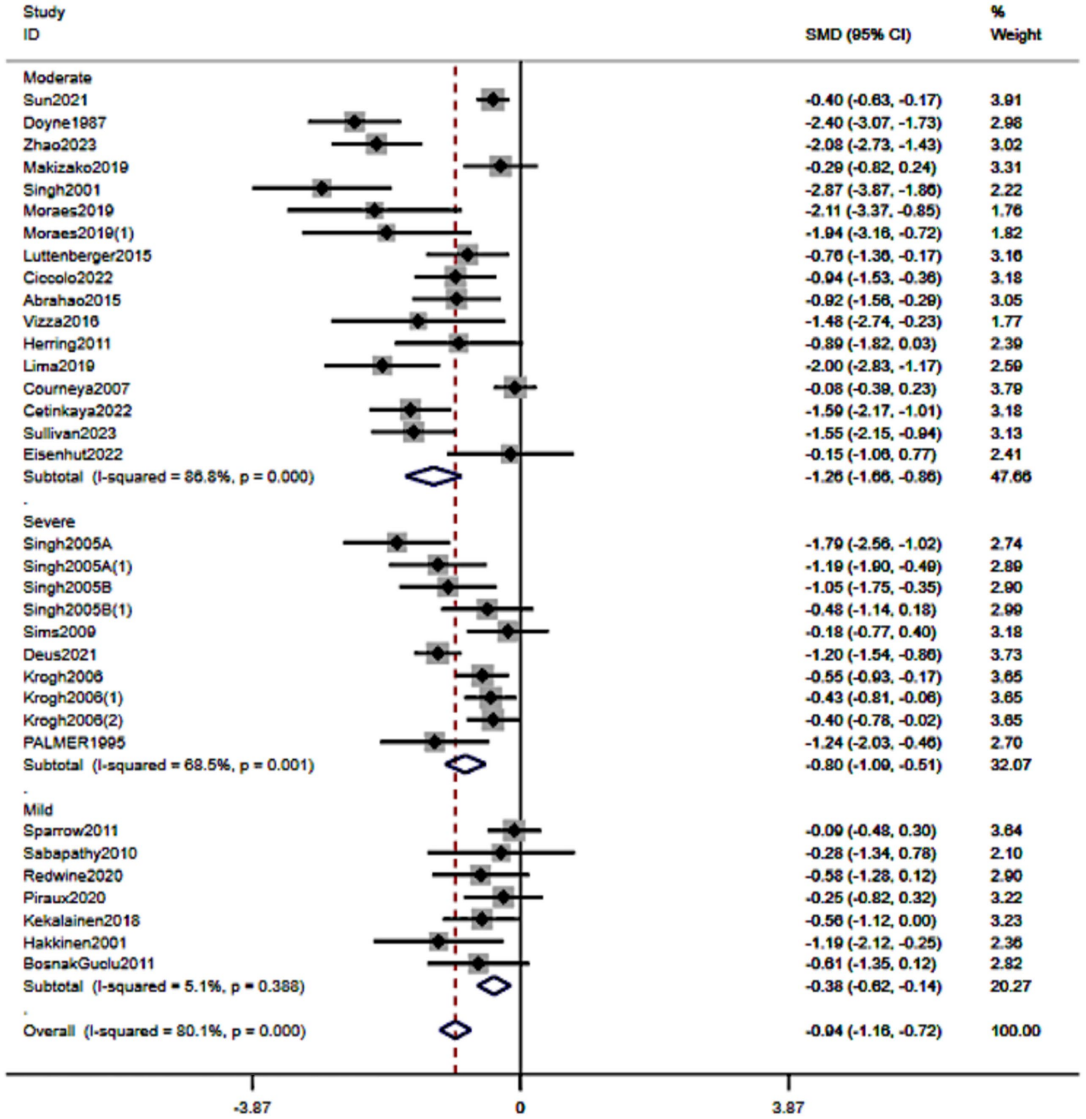
Forest plot of subgroup analyses stratified by depression severity. Weights are from random effects analysis.

When stratified by baseline severity (Figure 8), resistance training was associated with reduced depressive symptoms across categories: mild: SMD − 0.38 (95% CI − 0.62 to −0.14), moderate: −1.26 (−1.66 to −0.86), and severe: −0.80 (−1.00 to −0.51). Although point estimates differed, confidence intervals overlapped (notably between moderate and severe), and we did not conduct formal between-group tests. Overall, these findings indicate benefits across the severity spectrum, with any apparent differences interpreted cautiously.

#### 3.5.6 Training duration

**Figure 9:**
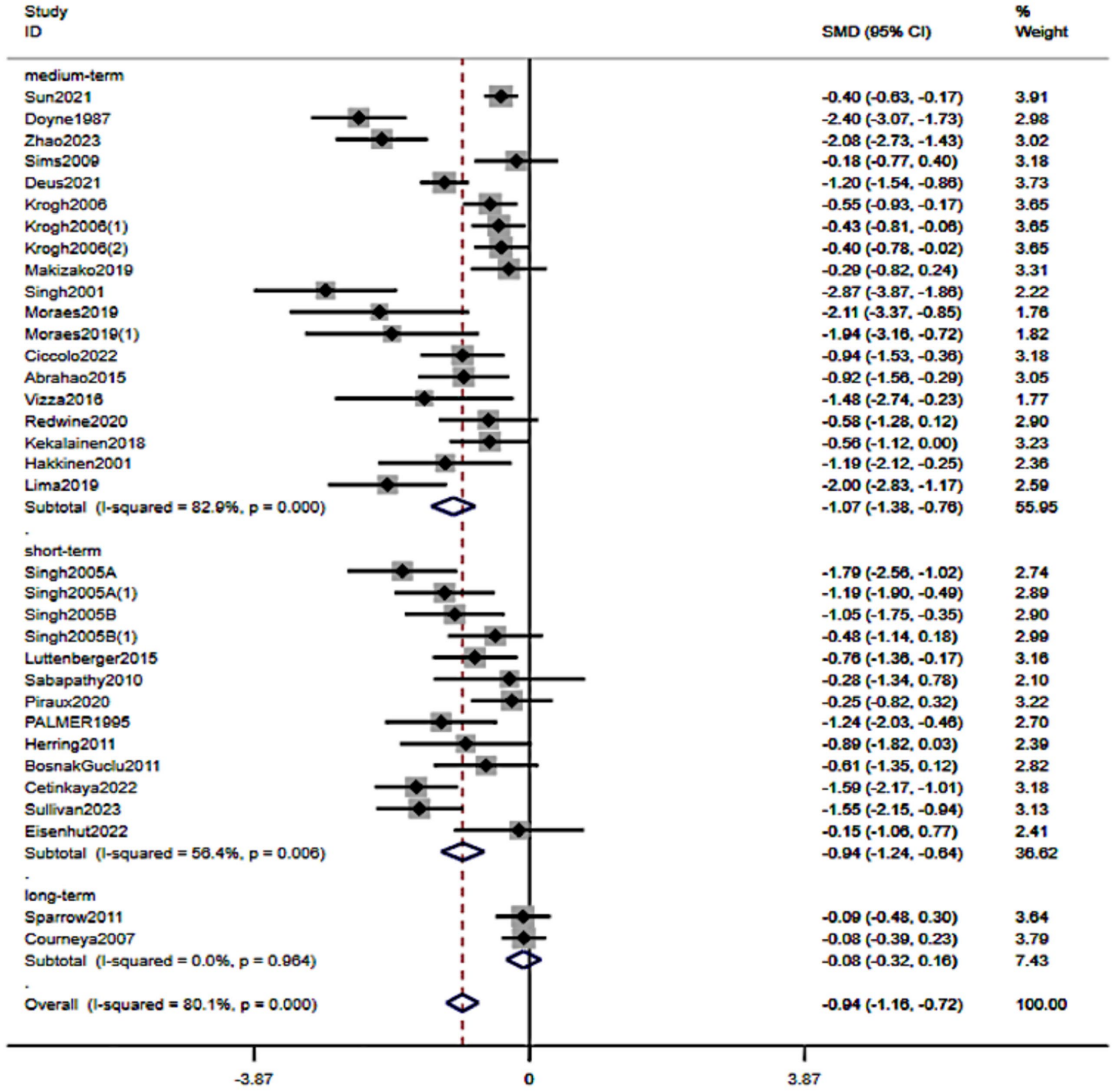
Forest plot of subgroup analyses stratified by training duration. Weights are from random effects analysis.

When stratified by program length, resistance training was associated with symptom reduction across several durations. Short-term interventions (4–8 weeks) showed a pooled effect of SMD − 0.94 (95% CI − 1.24 to −0.64), and medium-term interventions (9–24 weeks) SMD − 1.07 (−1.38 to −0.76); the confidence intervals overlapped, indicating broadly similar benefits. Estimates for ≥24 weeks were not statistically significant (*p* = 0.15) and imprecise. Overall, short-to-medium durations yielded clear improvements, whereas very long programs provided no additional, demonstrable advantage within the available evidence (Figure 9).

#### 3.5.7 Training intensity

**Figure 10:**
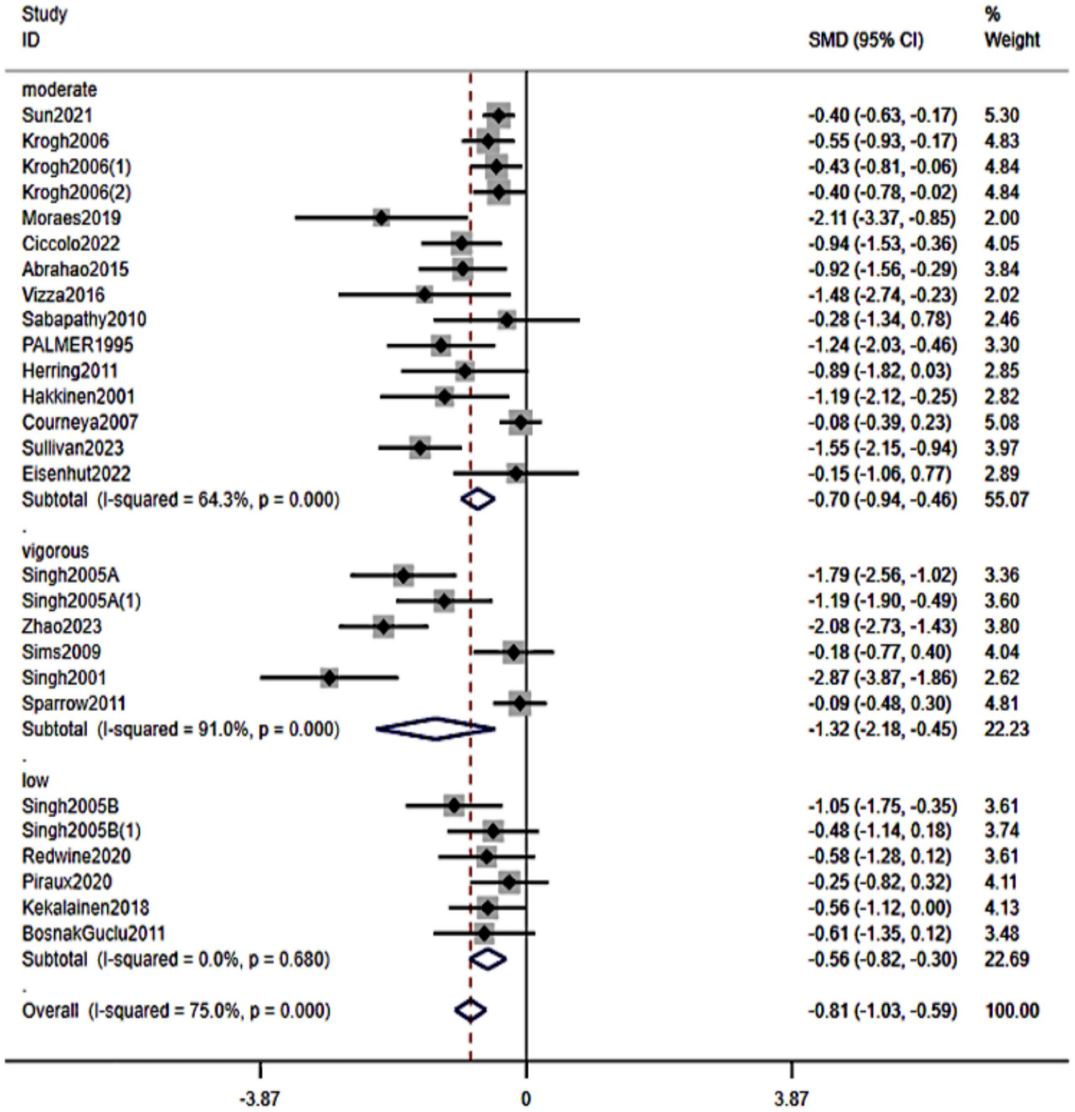
Forest plot of subgroup analyses stratified by intervention intensity. Weights are from random effects analysis.

We examined whether the intensity of resistance exercise (typically defined by the percentage of one-repetition maximum, 1RM) influenced the extent of symptom improvement (Figure 10). Data from 23 studies were categorized into low, moderate, and high intensity as per their protocols: Low-intensity RT (approximately ≤50% of 1RM): pooled SMD = −0.56 (95% CI: −0.82 to −0.30, *p* < 0.001). Moderate-intensity RT (around 50–75% of 1RM): pooled SMD = −0.70 (95% CI: −0.94 to −0.46, *p* < 0.001). High-intensity RT (≥75% of 1RM): pooled SMD = −1.32 (95% CI: −2.18 to −0.45, *p* < 0.001). All intensity strata showed significant antidepressant effects. Point estimates suggested a graded pattern—higher prescribed intensities yielded larger effects—while low and moderate intensities remained clearly beneficial for participants who may not train at high loads.

#### 3.5.8 Weekly intervention duration

**Figure 11:**
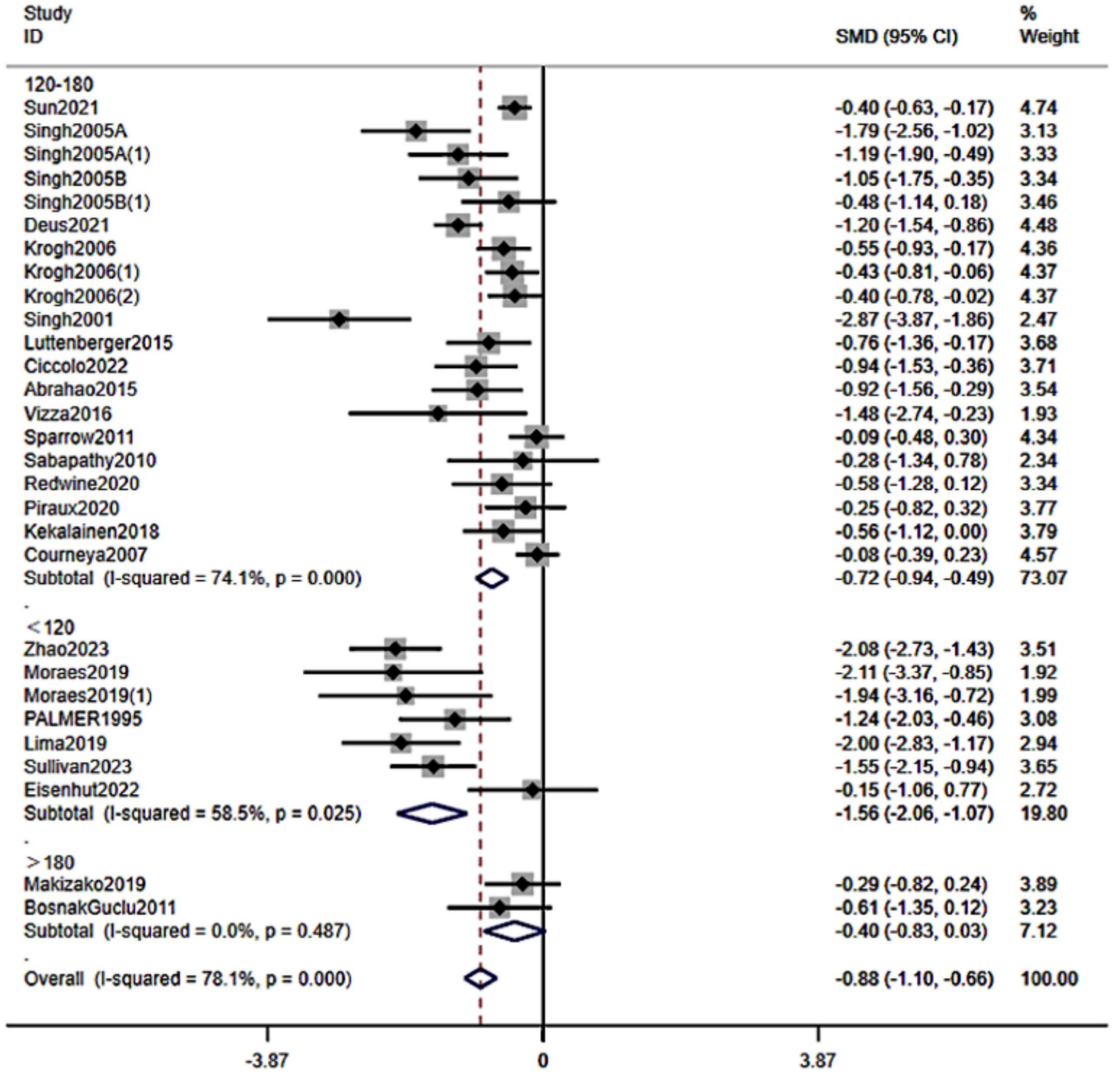
Forest plot of subgroup analyses stratified by length of intervention per week. Weights are from random effects analysis.

When stratified by weekly RT time (Figure 11), pooled effects were <120 min/week: SMD − 1.56 (95% CI − 2.06 to −1.07), 120–180 min/week: −0.72 (−0.94 to −0.49), and >180 min/week: not statistically significant (*p* = 0.07). Thus, ≤2 h/week and 2–3 h/week were both associated with meaningful symptom reductions, with the largest point estimate observed in the <120 min/week category.

### 3.6 Publication bias and sensitivity analyses

**Figure 12:**
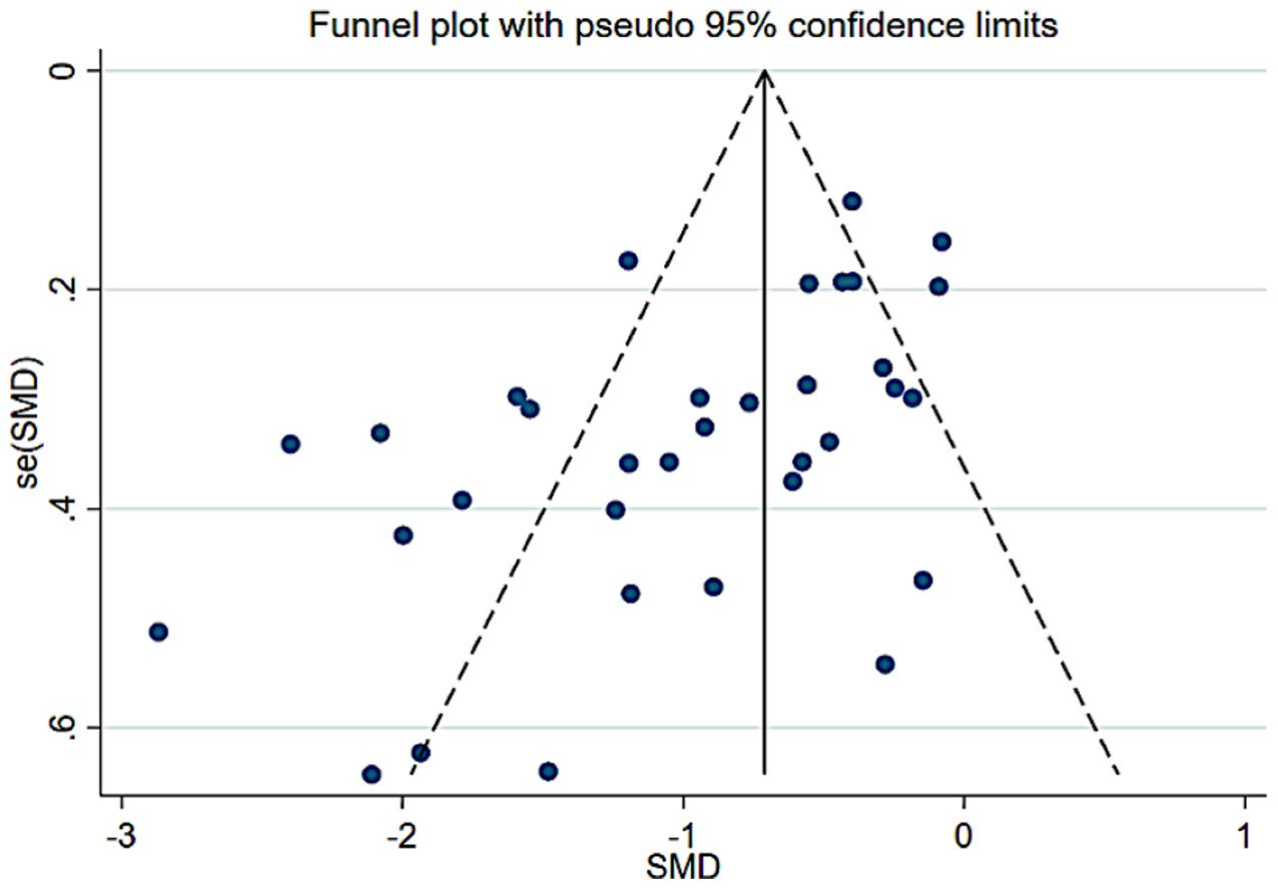
Funnel chart.

We evaluated the possibility of publication bias in our meta-analysis results. Visual inspection of the funnel plot for the primary outcome (Figure 12) revealed a roughly symmetrical distribution of study effects around the pooled estimate, with no obvious asymmetry that would suggest missing small studies with negative or null results. Begg’s test for small-study effects was non-significant (*p* > 0.05); however, given the study count and between-study heterogeneity, the power of these approaches is limited and undetected publication bias cannot be excluded (Table 2).

**Table 2:** Begg’s test.

Test method	Test statistic(Z)	*p*-value	Conclusion
Begg’s test	−3.14	1.9983	*p* > 0.05

**Figure 13:**
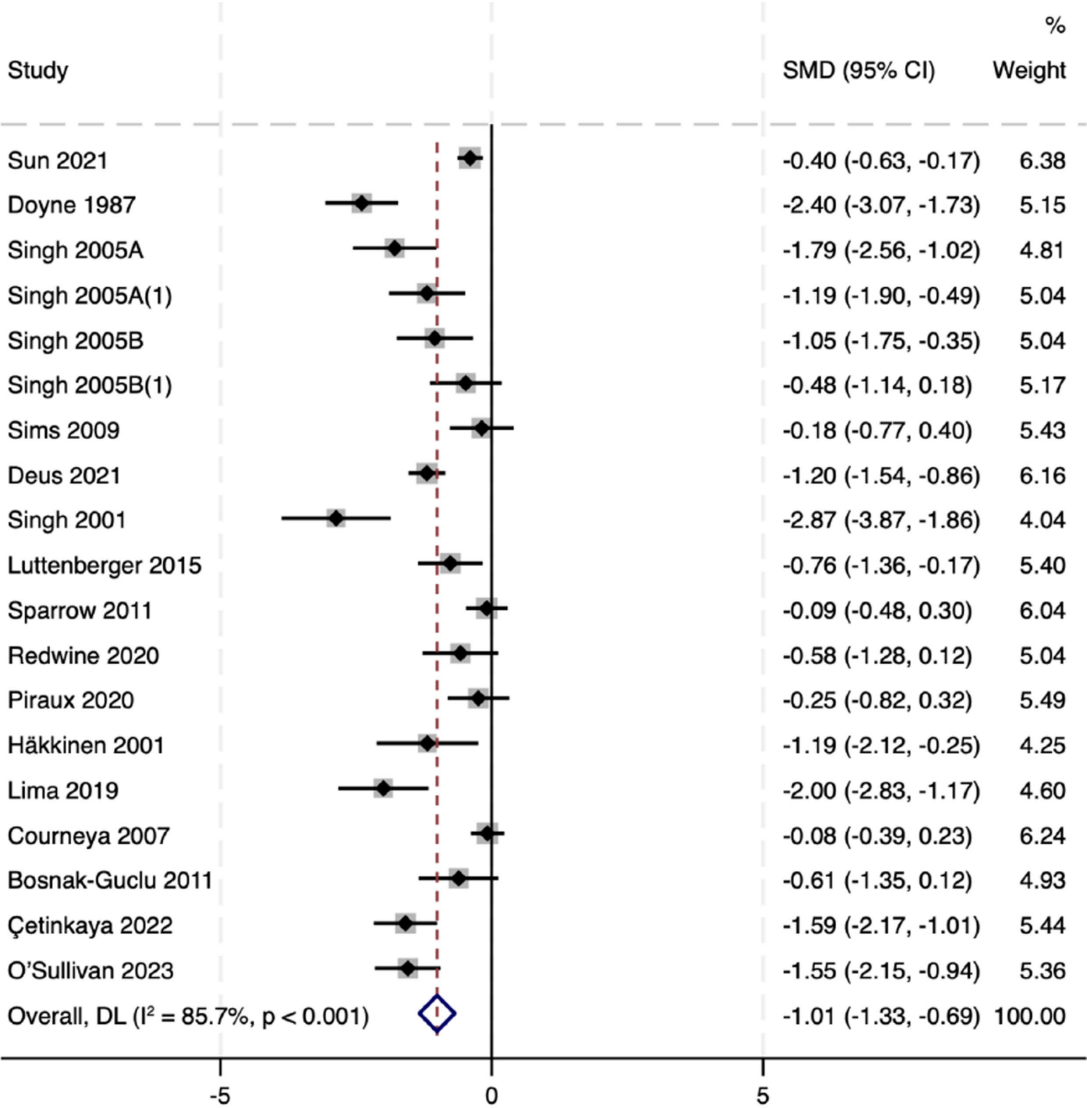
Forest plot after excluding high-risk trials. Weights are from random-effects model.

**Figure 14:**
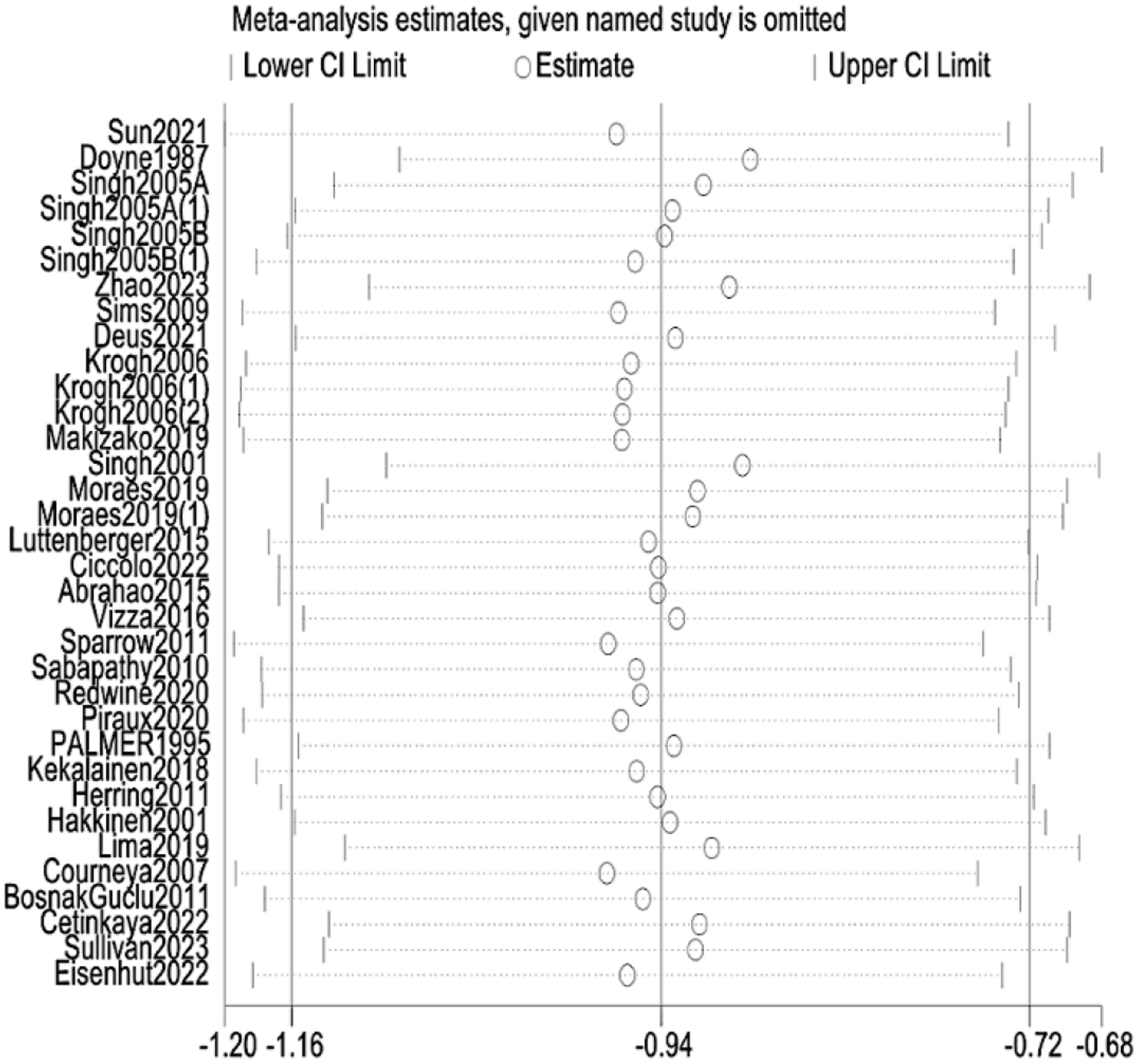
Sensitivity analysis.

Our sensitivity analysis demonstrated that the overall findings were robust. Sequentially removing each trial (one at a time) and re-running the meta-analysis did not yield any substantial change in the pooled SMD or its significance. In a quality-informed sensitivity analysis excluding high-risk trials (*k* = 19), the pooled effect remained significant and similar in magnitude (SMD − 1.01, 95% CI − 1.33 to −0.69; *I*^2^ = 85.7%; Figure 13). In leave-one-out analyses, pooled estimates ranged from SMD − 0.83 to −0.60, and all corresponding 95% CIs excluded the null. No single study unduly influenced the results; in all cases, the summary effect remained in favor of RT and within the CI range of the main analysis. This suggests that our conclusions are not driven by any outlier study and that the antidepressant effect of RT is a consistent finding across the body of evidence. The consistency of results upon study exclusion reinforces the reliability of the observed beneficial effect (Figure 14).

### 3.7 Summary of meta-analysis results

The meta-analysis showed that resistance training (RT) was associated with a significant reduction in depressive symptoms relative to control. Across key clinical and methodological contrasts, effects were broadly consistent. By clinical phenotype, benefits were observed in both primary depressive disorder and depression secondary to medical comorbidities, with a modest attenuation in the latter. By rater type, effects were directionally similar on self-report and observer-rated instruments. For the prespecified frequency moderator (<3 vs. ≥ 3 sessions/week), both strata demonstrated clear benefits without a compelling between-group difference.

Additional exploratory subgroup findings suggested improvements across age groups and across baseline severity categories (mild, moderate, severe). Regarding prescription characteristics, pooled estimates indicated benefits at low, moderate, and high intensities, with larger point estimates at higher prescribed intensities; and at <120 min/week and 120–180 min/week of weekly RT time, whereas >180 min/week did not show a statistically significant advantage. For program duration, short (4–8 weeks) and medium (9–24 weeks) interventions were beneficial, while ≥24 weeks did not demonstrate additional advantage within available data. Given multiplicity and unequal subgroup sizes, these moderator results are interpreted cautiously and regarded as hypothesis-generating.

Sensitivity analyses supported robustness: excluding high-risk trials yielded a pooled effect similar to the primary analysis, and leave-one-out checks showed no single study dominated the results. The funnel plot appeared broadly symmetric and Begg’s test was non-significant, though the power to detect small-study effects is limited. Overall, the findings indicate that RT confers meaningful antidepressant effects across diverse populations and implementations, while highlighting areas where more standardized, adequately powered trials are needed to refine dose-prescription guidance.

## 4 Discussion

This systematic review and meta-analysis synthesized evidence from 29 randomized controlled trials to clarify the efficacy of resistance training as a treatment for depression. The aggregated findings provide strong support that resistance training significantly alleviates depressive symptoms in adults, compared to no-exercise control conditions. This result aligns with and extends prior research on exercise and mental health, reinforcing that structured physical activity – and RT in particular – can play a valuable role in managing depression (Schuch and Vancampfort, 2021). Importantly, our analysis focused specifically on resistance exercise, filling a gap in the literature where most previous meta-analyses had emphasized aerobic exercise. We found a large overall effect size (SMD ~ −0.94), suggesting that RT can be at least as effective as, if not more effective than, many other lifestyle or psychosocial interventions for depression. These findings add to a growing recognition that exercise modalities targeting muscular strength are effective and should be considered in clinical recommendations for depression alongside aerobic exercise.

Potential Mechanisms: The beneficial effects of resistance training on mood and depressive symptoms likely arise from a combination of biological, psychological, and social mechanisms. Biologically, RT induces a cascade of neurochemical and physiological changes that can counteract depression. Regular RT has been shown to modulate key neurotransmitters and neuromodulators – increasing the availability of serotonin and dopamine and promoting the release of endorphins – which are associated with improved mood. RT also stimulates neuroplasticity (e.g., via increased brain-derived neurotrophic factor, BDNF) and reduces systemic inflammation and cortisol levels, thereby mitigating some of the neuroendocrine stress responses linked to depression (Schuch and Vancampfort, 2021). Improvements in metabolic health and insulin sensitivity from RT may further relieve depressive symptoms, as metabolic dysregulation has been implicated in depression pathophysiology.

Psychologically, engaging in RT can enhance self-efficacy and self-esteem by providing a sense of mastery and accomplishment as individuals gain strength and functional improvements (Braith and Stewart, 2006). Many depressed patients struggle with feelings of helplessness or poor self-image; seeing tangible progress (such as lifting heavier weights or improving physical performance) can directly improve one’s body image and confidence. This boost in self-worth and perceived control over one’s body translates into better overall mental health. Moreover, RT can serve as a positive coping strategy or structured activity that occupies time and attention, potentially reducing rumination and depressive thought patterns.

Socially, participating in resistance training – particularly in group exercise classes or under the guidance of a trainer – offers opportunities for social interaction and support (Xie et al., 2021). The social engagement aspect of exercise can reduce feelings of isolation and loneliness that often accompany depression. Group RT settings may foster a sense of camaraderie and accountability among participants, while supervised sessions provide encouragement and human connection with instructors or therapists. These social factors can be therapeutic in their own right. In sum, resistance training addresses depression on multiple levels: physiologically improving brain and body health, psychologically empowering the individual, and socially connecting them with others, all of which are critical dimensions for recovery from depression.

Subgroup analyses in our meta-analysis yielded important insights into moderators of RT effectiveness, which have practical implications for tailoring interventions.

In particular, we examined whether the antidepressant impact of RT varied by clinical phenotype (primary vs. comorbid depression), rater type (self-report vs. observer-rated), and key prescription features (notably training frequency), with additional exploratory analyses for age, baseline severity, program duration, intensity, and weekly volume.

### 4.1 Participant characteristics

#### 4.1.1 Mpact of clinical phenotype

Across clinical phenotypes, RT was associated with improvements in depressive symptoms, with a tendency toward larger benefits in primary depressive disorder than in depression secondary to medical comorbidities. Several mechanisms may account for this gradient: higher somatic burden and symptom complexity in comorbid populations can limit achievable training loads and progression; concurrent treatments and rehabilitation may dilute incremental effects; and disease-related fatigue or mobility constraints can reduce adherence and realized dose (Gordon et al., 2018).

Clinically, comorbidity should prompt adaptation rather than exclusion. Programs may emphasize conservative load progression, symptom-contingent adjustments, integration with medical rehabilitation schedules, and enhanced supervision/adherence support to optimize exposure and safety (Barker et al., 2023). Future work would benefit from prospective phenotype stratification, comorbidity-aware prescription algorithms, and transparent reporting of interaction tests to clarify how medical complexity shapes response to RT (Riebe et al., 2015).

#### 4.1.2 Influence of age

This meta-analysis supports that resistance training improves depressive symptoms across young, middle-aged, and older adults, extending the potential applicability of RT. Apparent age-related differences were observed, with effects appearing larger in younger adults, and several factors may contribute. Younger individuals generally have greater physiological resilience and capacity for higher relative intensities; they often tolerate more strenuous RT and recover more quickly, which may enhance neuromuscular and neurobiological adaptations relevant to mood (e.g., endorphins, monoamines) (Kvam et al., 2016; Gordon et al., 2018). Adaptations of the developing adult brain and body may also contribute to stronger mood responses.

Moreover, younger adults may derive particular psychological and social benefits from RT. They are often motivated by the social and competitive aspects of working out – e.g., going to the gym with friends, tracking personal records, or seeing tangible improvements in appearance and strength – which can boost enjoyment and adherence to the program (Stonerock et al., 2015). This age group might integrate exercise as part of their identity or routine more readily, resulting in sustained engagement. The sense of achievement from mastering new exercises or lifting progressively heavier weights likely contributes to improved self-esteem and reduction in depressive symptoms for younger participants (Gordon et al., 2018). Social interaction during training sessions (in group classes or gym settings) can also be especially valuable for younger individuals, who may be particularly sensitive to social connectivity as a support against depression.

For middle-aged and older adults, benefits were also evident, though effect sizes may be somewhat smaller, potentially reflecting comorbidities and lower baseline fitness. RT can improve strength, function, and fatigue in these populations, changes that can translate to better daily functioning and mood (Danielsson et al., 2013). Given potentially slower recovery, longer interventions may help accumulate benefit; our subgroup analysis suggested that medium-term programs were effective across ages. In practice, moderate intensities, gradual progression, and higher supervision are common in older adults, balancing safety and dose delivery. Older participants in the included trials still achieved meaningful symptom reductions, and the structure/social interaction of sessions may also confer psychological gains (Mammen and Faulkner, 2013). Tailoring dose and progression to comorbidity and capacity is advisable, and some individuals may require longer training periods to realize comparable benefits (Harvey et al., 2018).

In summary, age does not appear to limit the clinical use of RT for depression. Programs can be individualized—emphasizing adequate stimulus and progression in younger adults, and safety, adherence, and functional goals in middle-aged/older adults—to support both mood and physical outcomes.

#### 4.1.3 Effect of depression severity

Subgroup analysis indicated that resistance training (RT) had the greatest impact on patients with moderate depression. This may be explained by the fact that individuals with moderate symptoms have greater capacity for improvement compared to those with mild depression, yet do not face the extensive physiological and psychological challenges associated with major depression (Gordon et al., 2018). Research suggests that patients with moderate depression tend to experience faster positive feedback from physical activity, such as mood enhancement, improved sleep, and reduced anxiety (Stanton and Reaburn, 2014).

In contrast, individuals with major depression may require more comprehensive interventions, including pharmacotherapy and psychological therapy. While RT can offer supplementary benefits, it is often insufficient as a standalone treatment for this population (Schuch et al., 2016). Patients with major depression frequently encounter complex neurochemical imbalances and a sense of helplessness, which may impede immediate benefit from exercise interventions (Stanton and Reaburn, 2014). Accordingly, a multimodal treatment approach is generally recommended to achieve optimal outcomes.

For patients with mild depression, the potential for improvement is typically limited by the less severe nature of their symptoms, which may often be managed with lifestyle modifications (Schuch and Stubbs, n.d.). While RT is still beneficial, the degree of improvement observed is generally smaller than that seen in individuals with moderate depression (Mammen and Faulkner, 2013).

### 4.2 Optimal training parameters

#### 4.2.1 Impact of frequency

Across the included trials, antidepressant benefits were observed at both lower (<3 sessions/week) and higher (≥3 sessions/week) training frequencies. Consistent with our exploratory subgroup framework, we did not detect a compelling between-frequency difference; nevertheless, from an implementation standpoint, scheduling resistance training on roughly 3 days per week provides a practical balance between sufficient stimulus, recovery, and adherence, and aligns with prior exercise-prescription literature. In practice, weekly frequency should be coordinated with intensity and total volume (e.g., session length and load progression) so that progression is achievable without excessive fatigue, with supervision used to support technique, safety, and compliance (Gordon et al., 2018; Danielsson et al., 2013; Khodadad Kashi et al., 2023).

#### 4.2.2 Impact of intensity

Antidepressant improvements were observed across low (≈ ≤ 50% 1RM), moderate (≈50–75% 1RM), and high (≥75% 1RM) intensities, with a pattern suggesting larger effects at higher loads. Mechanistically, heavier training is associated with greater neuromuscular stimulus and may amplify mood-relevant pathways (e.g., monoamines, endorphins, neurotrophic signaling) (Gordon et al., 2018; Mammen and Faulkner, 2013; Harvey et al., 2018). In practice, intensity should be progressed gradually and paired with appropriate supervision to ensure technique, safety, and adherence; when high loads are not feasible, moderate-intensity protocols with planned progression remain reasonable and effective.

#### 4.2.3 Impact of duration

Across trials, programs of roughly 9–24 weeks tended to yield the most consistent improvements in depressive symptoms, plausibly reflecting the time needed to accrue neuromuscular adaptation, behavioral routine, and mood-relevant neurobiological changes. Very brief interventions can still help but may provide an insufficient exposure window, whereas extending programs beyond ~24 weeks did not consistently add benefit—likely a function of adherence challenges and plateauing gains rather than a true lack of efficacy (Schuch et al., 2016; Schuch and Stubbs, n.d.). In practice, planning for a medium-term cycle with built-in progression and retention strategies appears pragmatic (Gordon et al., 2018).

#### 4.2.4 Impact of weekly volume

Benefits were observed at modest weekly durations (≈ < 120 to 120–180 min/week), with higher volumes not reliably conferring additional advantage. This aligns with the idea that effective “dose” can be achieved by combining session frequency and intensity without excessive weekly minutes, which may also support adherence (Stanton and Reaburn, 2014; Schuch et al., 2016; Biddle and Asare, 2011). When feasible, distributing work across multiple sessions while moderating per-session length can help deliver sufficient stimulus and limit fatigue, particularly alongside progressive loading.

## 5 Limitations

Several limitations warrant consideration. First, substantial between-study heterogeneity remained despite random-effects modeling. Sources likely include clinical mix (primary depressive disorder vs. depression secondary to medical comorbidities), differences in rater type (self-report vs. observer-rated instruments) and in instrument constructs (e.g., tools tailored to older adults), as well as variability in supervision/adherence and concomitant treatments. While standardized mean differences harmonize scale metrics, they do not eliminate construct heterogeneity; clinical interpretability was therefore framed qualitatively rather than via precise back-translation to specific scales.

Second, subgroup and moderator analyses were prespecified as exploratory and no formal multiplicity correction was applied. Several strata had small study counts (k < 5), yielding imprecise estimates and increasing the risk of chance findings. These contrasts should be viewed as hypothesis-generating and confirmed in adequately powered, preregistered trials.

Third, the operationalization of training prescription varied across trials (e.g., intensity defined by %1RM vs. RPE; diversity in progression rules and supervision), and some studies incompletely reported key parameters. Although we conducted sensitivity checks (e.g., frequency strata; exclusion of unreported categories where applicable), imprecision in exposure classification limits the firmness of “optimal prescription” inferences.

Fourth, risk of bias considerations persist. Blinding of participants/personnel is rarely feasible in exercise trials and may introduce expectancy or performance/detection biases, potentially inflating effects. Quality-informed sensitivity analyses (excluding high-risk trials; leave-one-out) yielded similar magnitudes, but residual bias cannot be ruled out.

Fifth, most trials assessed post-intervention outcomes only, limiting conclusions about durability and real-world maintenance. Future studies should incorporate preregistered follow-ups (e.g., 3–12 months) with adherence tracking and maintenance strategies.

Sixth, small-study/publication bias cannot be excluded. The funnel plot appeared broadly symmetric and Begg’s test was non-significant, but statistical power to detect asymmetry is limited with this study count and heterogeneity.

Seventh, although multi-arm trials were handled to avoid unit-of-analysis errors (combining intervention arms or splitting shared controls), residual differences in co-interventions, settings, and healthcare systems constrain generalizability. Regional patterns, when observed, are context-dependent and not interpreted as causal.

Collectively, these limitations argue for rigorously reported, multicenter RCTs using standardized intervention checklists (e.g., TIDieR/CERT), clearer prescription definitions and fidelity/adherence metrics, preregistration of subgroup hypotheses, and longitudinal follow-up to refine effect estimates and implementation guidance.

## 6 Conclusion

This meta-analysis indicates that resistance training (RT) is associated with clinically meaningful reductions in depressive symptoms across diverse adult populations. Signals from exploratory subgroup analyses suggest that programs delivered at approximately three sessions per week, over 9–24 weeks, and at higher relative intensities may be linked to larger improvements, with a total weekly RT time around 2 h; however, these contrasts were not multiplicity-adjusted and should be regarded as preliminary rather than prescriptive.

From a practice standpoint, RT can be considered a viable, accessible adjunct to standard depression care, implemented with attention to individual capacity, medical comorbidity, supervision, and adherence. Given residual heterogeneity and the challenges of blinding in exercise trials, clinical interpretation should remain cautious, and programs should be tailored rather than one-size-fits-all. Future preregistered, multicenter RCTs with standardized reporting (e.g., TIDieR/CERT) and longer follow-up are needed to refine dose–response guidance and clarify durability of benefits.

## Data Availability

All data and material reported in this review and meta-analysis were from peer reviewed publications. The datasets supporting the conclusions of this article are included within the article and its additional files.
