# Supplementary material for: Resistance training for depression: a systematic review and meta-analysis of randomized controlled trials
Source: Front Psychol. 2025 Dec 15;16:1655855. doi: 10.3389/fpsyg.2025.1655855 (PMC12745427; doi:10.3389/fpsyg.2025.1655855)
Supplement: Supplementary file 1 [file Supplementary_Table_1.DOCX]

**Table S1. Complete PubMed search strategy.**

| Database | Final search date | Full search string |
| --- | --- | --- |
| PubMed (MEDLINE) | 10 Oct 2025 | ( "Resistance Training"[Mesh] OR "Weight Lifting"[Mesh] OR "resistance training"[tiab] OR "progressive resistance"[tiab] OR "resistance exercise*"[tiab] OR "strength training"[tiab] OR "strength exercise*"[tiab] OR "weight lifting"[tiab] OR "weight-lifting"[tiab] OR "weight-bearing exercise*"[tiab] OR "weight-bearing training"[tiab] OR "Exercise Therapy"[Mesh] OR "exercise therapy"[tiab] ) AND ( "Depressive Disorder"[Mesh] OR "major depressive disorder"[tiab] OR "major depression"[tiab] OR depression[tiab] OR "depressive disorder*"[tiab] OR "depressive symptom*"[tiab] OR "depressive syndrome*"[tiab] OR "unipolar depression"[tiab] OR melancholia[tiab] OR "neurotic depression"[tiab] OR "mild depression"[tiab] OR "moderate depression"[tiab] OR "severe depression"[tiab] ) AND ( randomized controlled trial[pt] OR controlled clinical trial[pt] OR randomized[tiab] OR randomised[tiab] OR randomly[tiab] OR trial[ti] OR groups[tiab] OR placebo[tiab] ) NOT (animals[mh] NOT humans[mh]) |
